# Peroxidasin enables melanoma immune escape by inhibiting natural killer cell cytotoxicity

**DOI:** 10.1002/1878-0261.70191

**Published:** 2026-01-28

**Authors:** Hsu‐Min Sung, David Bickel, Lena C. M. Krause, Daria Ezeriņa, Christian Ickes, Julian Wojtachnia, Christine S. Gibhardt, Magdalena Shumanska, Khadija Wahni, Andrea Paluschkiwitz, Julia Malo Pueyo, Ekaterina Baranova, Wim Vranken, Hedwig Stanisz, Ioana Stejerean‐Todoran, Michael P. Schön, Joris Messens, Ivan Bogeski

**Affiliations:** ^1^ Molecular Physiology HKP, University Medical Center Göttingen Germany; ^2^ VIB‐VUB Center for Structural Biology VIB Brussels Belgium; ^3^ Brussels Center for Redox Biology Brussels Belgium; ^4^ Structural Biology Brussels Vrije Universiteit Brussel Brussels Belgium; ^5^ Interuniversity Institute of Bioinformatics in Brussels ULB‐VUB Brussels Belgium; ^6^ Department of Dermatology, Venereology and Allergology University Medical Center Göttingen Germany

**Keywords:** AlphaFold, immunotherapy, melanoma, molecular dynamics simulations, NK cells, peroxidasin, redox, tumor microenvironment

## Abstract

Peroxidasin (PXDN), an extracellular matrix (ECM)‐associated peroxidase, has been implicated in cancer progression. However, its roles in melanoma biology and therapeutic sensitivity remain unclear. Here, we demonstrate that elevated *PXDN* expression is associated with poor prognosis and reduced survival in melanoma patients. Functional studies revealed that *PXDN* depletion impairs melanoma cell proliferation, disrupts the cell cycle, and reduces melanoma cell invasive capacities. Furthermore, we found that secreted PXDN modulates anti‐melanoma immunity by enhancing melanoma resistance to natural killer (NK)‐cell‐mediated cytotoxicity. Structural modeling identified a trimeric organization of PXDN, stabilized by disulfide‐linked peroxidase domains. Molecular dynamics simulations identified a previously unknown inhibitory interaction between the PXDN N‐terminal leucine‐rich repeat domain and the NK cell‐activating receptor NKG2‐D type II integral membrane protein (NKG2D). These findings uncover a redox‐independent role for PXDN in promoting immune evasion and tumor progression. Overall, our study highlights PXDN as a critical regulator of melanoma cell biology and a potential therapeutic target for NK‐cell‐based immunotherapy in melanoma and other solid cancers.

## Graphical abstract

Peroxidasin (PXDN) is secreted by melanoma cells and binds the NK cell receptor NKG2D, thereby suppressing NK cell activation and cytotoxicity. PXDN depletion restores NKG2D signaling and enables effective NK cell–mediated melanoma killing. These findings identify PXDN as a previously unrecognized immune evasion factor and a potential target to improve NK‐cell‐based immunotherapy.

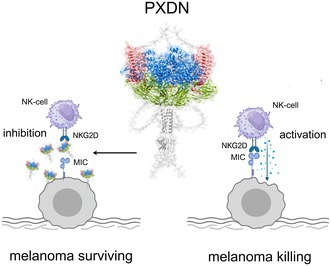

**Abbreviations**
ACTB: beta‐actin
BSA: Bovine Serum Albumin
CD48: CD48 antigen–B‐lymphocyte activation marker BLAST‐1 (P09326)
CD70: CD70 antigen–Tumor necrosis factor ligand superfamily member 7 (P32970)
CD137: CD137 antigen–Tumor necrosis factor receptor superfamily member 9 isoform X1 (Q07011)
DEG: differentially expressed gene
DGE: differential gene expression
DMEM: Dulbecco's modified Eagle's medium
DTT: dithiothreitol
ECM: extracellular matrix
EhD‐1: ethidium homodimer‐1
EMT: epithelial‐mesenchymal transition
FCS: fetal calf serum
FBS: fetal bovine serum
FOX: Ferrous Oxidation in Xylenol
GAPDH: glyceraldehyde‐3‐phosphate dehydrogenase
GEO: Gene Expression Omnibus
GO: gene ontology
GSH: glutathione
IDT: integrated DNA technologies
IHC: immunohistochemistry
KD: knockdown
Log2FC: Log2 fold change
LRR: leucine‐rich repeats
MD: molecular dynamics
MFI: mean fluorescence intensity
MG50: melanoma gene 50
MM‐GB/SA: Molecular Mechanics/Generalized Born Surface Area
NAC: N‐acetyl cysteine
NK: natural killer
NKG2D: NKG2‐D type II integral membrane protein (A4GHD0)
*NKmK*
: NK cell–mediated killing
NR: non‐responder
OCR: oxygen consumption rates
PBMC: peripheral blood mononuclear cell
PBS: phosphate‐buffered saline
*P*adj: adjusted *P*‐value
p‐H3: phospho‐histone H3
pNK: primary NK
PAE: predicted aligned error
PAGE: polyacrylamide gel electrophoresis
PBS: phosphate‐buffered saline
PFA: paraformaldehyde
pLDDT: predicted local distance difference test
PMSF: phenylmethylsulfonyl fluoride
PXDN: peroxidasin
R: responders
RGP: radial growth phase
RNP: ribonucleoprotein
RMSD: root mean square deviation
SDS: sodium dodecyl sulfate
SKCM: skin cell melanoma
TBP: TATA box binding protein
TBS: Tris‐buffered saline
TCA: trichloroacetic acid
TIGER: Tumor Immunotherapy Gene Expression Resource
TNR9: tumor necrosis factor receptor superfamily member 9 (Q07011)
TPM: transcripts per million
TRAIL‐R2: tumor necrosis factor receptor superfamily member 10B isoform 1 precursor (Q9QZM4)
TrxR1: thioredoxin reductase 1
VGP: vertical growth phase
VPO1: vascular peroxidase‐1
WT: wild‐type

## 1 Introduction

Melanoma is among the most aggressive and lethal skin cancers, with a 5‐year survival rate below 35% for patients with distant metastases [1]. Over the past decade, two major treatment strategies have emerged: targeted therapies that inhibit frequently activated BRAF and MEK kinases [2], and immune checkpoint inhibitors targeting CTLA4 and the PD1–PDL1 axis [3, 4]. While these therapies have significantly improved outcomes, melanoma's high heterogeneity and plasticity often lead to drug resistance, posing a persistent clinical challenge [5, 6].

Recent studies have highlighted the role of natural killer (NK) cells in antitumor immunity across various cancers, including melanoma [7, 8, 9, 10, 11]. The presence of tumor‐infiltrating NK cells correlates with improved survival in melanoma patients [12, 13], yet NK cell‐based therapies have not reached clinical applications in solid tumors. Our previous work identified a protein signature predicting melanoma susceptibility to NK cell‐mediated cytotoxicity and patient response to CTL‐based therapies like nivolumab [7]. Other screens have identified additional regulators of NK cell responses [14, 15, 16], but the mechanisms controlling NK–melanoma interactions remain poorly understood. Identifying new molecular regulators of NK cell cytotoxicity could accelerate NK cell‐based therapeutic development.

Melanoma also relies heavily on antioxidant systems to counter oxidative stress [17, 18, 19, 20]. Antioxidant treatments such as N‐acetyl cysteine (NAC) can paradoxically promote invasion and metastasis [21, 22], while targeting antioxidant enzymes like thioredoxin reductase 1 (TrxR1) selectively kills cancer cells [23, 24], highlighting redox regulators as potential therapeutic targets.

Peroxidasin (PXDN), also known as melanoma gene 50 (MG50) or vascular peroxidase‐1 (VPO1), was initially identified as a melanoma antigen recognized by cytolytic T lymphocytes [25]. PXDN is a heme‐binding peroxidase featuring five N‐terminal leucine‐rich repeats (LRR) and a C‐terminal peroxidase domain [26]. PXDN is ER‐localized and secreted into the extracellular matrix (ECM) via an N‐terminal signal peptide [27]. Its expression is regulated by the redox‐sensitive transcription factor Nrf2 [28] and epithelial‐mesenchymal transition (EMT) regulator Snail [29]. PXDN catalyzes sulfilimine bond formation in collagen IV which is crucial for ECM assembly [30, 31]. Conversely, elevated PXDN levels have been linked to tumor progression, oxidative stress resistance, and apoptosis suppression in multiple cancers [32, 33]. PXDN depletion impairs melanoma invasion [34, 35], and pan‐cancer studies support its potential as an immunotherapy target and diagnostic marker [36, 37, 38]. However, the exact mechanisms by which PXDN contributes to melanoma progression and immune evasion remain unclear.

In this study, we demonstrate that PXDN is upregulated in melanoma cells from distal metastases and is inversely correlated with patient survival. PXDN promotes melanoma cell proliferation, invasion, and survival *in vitro*. Using real‐time NK cell cytotoxicity assays, we uncover a novel, peroxidase‐independent role of PXDN in suppressing NK cell‐mediated killing (*NKmK*). Structural modeling with AlphaFold2_multimer followed by molecular dynamics simulations suggest that PXDN interacts with NK cell surface receptor NKG2D via its N‐terminal LRR domain l, thereby inhibiting *NKmK*. Collectively, our findings identify PXDN as a prognostic biomarker and promising therapeutic target for advanced melanoma.

## 2 Materials and methods

### 2.1 Cell culture and reagents

All melanoma cell lines (WM35: CVCL_0580, WM1366: CVCL_6789, WM793: CVCL_8787, WM9: CVCL_6806, WM983B: CVCL_6809, WM3682: CVCL_AP78, WM858: CVCL_C283, WM1026: CVCL_AP84, WM88: CVCL_6805, WM3918: CVCL_C279, WM164: CVCL_7928, 1205Lu: CVCL_5239, WM3734: CVCL_6800) were kindly provided by Prof. Meenhard Herlyn (Wistar Institute, Philadelphia, PA, USA). Melanoma cell lines were maintained in Dulbecco's modified Eagle's medium (DMEM), high glucose (Gibco™, Waltham, MA, USA; #11965092) supplemented with 10% fetal bovine serum (FBS, Gibco). All cell lines were cultured at 37 °C in 5% CO_2_ and were consistently tested negative for Mycoplasma using the PCR Mycoplasma Test Kit I/C (PromoKine, #PK‐CA91‐1048; Heidelberg, Germany), following the manufacturer's instructions. Melanoma cell lines were authenticated by short tandem repeat (STR) profiling within the last 3 years. For primary NK cell isolation, leukocyte reduction system chambers were provided by the local blood bank of the Institute of Transfusion Medicine, University Medical Centre Göttingen (UMG). The experiments were undertaken with the understanding and written consent of each donor and the study methodologies were approved by the local ethics committee (UMG ethics approval number: 2/3/18). Peripheral blood mononuclear cells (PBMCs) were isolated from healthy donors in the period between September 2019 and March 2022 at the University Medical Centre Göttingen by density gradient centrifugation using LeucoSep tubes (50 mL, Greiner Bio‐One: Frickenhausen, Germany, #277290) and Lymphocyte Separation Medium 1077 (PromoCell: Heidelberg, Germany, #C‐44010). pNK cells were isolated from PBMCs by negative bead isolation using the NK Cell Isolation Kit, human (Miltenyl Biotec, #130–092‐657). Isolated pNK cells were maintained in AIMV medium (Life Technologies, #12055–091; Carlsbad, CA, USA) supplemented with 10% fetal calf serum (FCS) (Sigma‐Aldrich, Darmstadt, Germany; #A9418) and 0.05 mg·mL^−1^ IL2 (Thermo Fisher Scientific, #15596026) for 60–72 h. The leukemia cell line K562 (ATCC, #CCL‐243, RRID: CVCL_0004) was cultured in RPMI1640 medium (Thermo Fisher Scientific, #21875–034) supplemented with 10% FCS. To increase experimental consistency, all cell lines were passaged under sub‐confluent cell density and were kept in culture for less than 3 months (24–30 passages) before a new batch of cryotube was thawed. All reagents were purchased from Sigma‐Aldrich if not specified otherwise.

### 2.2 Bioinformatic analysis

#### 2.2.1 Identification of candidate redox regulator

To identify genes encoding redox‐regulating proteins showing distinct expression between healthy melanocyte and malignant melanoma, 16 Gene Expression Omnibus (GEO) datasets of microarray analysis were downloaded from NCBI (https://www.ncbi.nlm.nih.gov/geo/) for differential gene expression (DGE) analysis. 29 DGE profiles were generated by group comparisons (melanocyte versus melanoma). GEO accession numbers of the datasets used in this study and the information of sample grouping are listed in Table S1. DGE profiles including Log_2_ fold change (Log_2_FC) and adjusted *P*‐value (*P*adj) were calculated by the web‐based bioinformatic tool, GEO2R analysis (https://www.ncbi.nlm.nih.gov/geo/geo2r/). Log_2_FC and *P*adj of 83 selected human redox regulators were extracted from the GEO2R analysis for the calculation of regulation score.

#### 2.2.2 Kaplan–Meier survival estimation

To generate survival plots, melanoma patient datasets (SKCM‐TCGA) were downloaded from cBioPortal on 26.08.2020. Kaplan–Meier plots were calculated using lifelines [39]. Patients were divided into patients with either low or high *PXDN* expression by scanning 20–80% of *PXDN* expression value and determining the optimal separation in terms of survival based on a log‐rank test. For detailed analyses, patients were separated by different melanoma tumor stages (0–II and III–IV), by BRAF and NRAS wild‐type or mutant. For patients treated with immunotherapies, survival estimation plot was adapted from the Tumor Immunotherapy Gene Expression Resource (TIGER; http://tiger.canceromics.org/) [40]. *P‐*values were calculated by using univariate Cox regression analysis.

#### 2.2.3 PXDN expression comparison

Melanoma patient datasets were evaluated based on differences between *BRAF* and *NRAS* wild‐type and mutant or progression and remission. Data was derived by the TCGA Research Network: https://www.cancer.gov/tcga/ on 07.09.2021. FPKM normalized expression values were converted to transcripts per million (TPM). Patients were separated again according to their status and *P*‐values were calculated by using the Mann–Whitney U‐test. For patients treated with immunotherapies, box plots illustrating *PXDN* expression among responders and non‐responders were adapted from the Tumor Immunotherapy Gene Expression Resource (TIGER; http://tiger.canceromics.org/) [40]. *P*‐values were calculated by the Wilcoxon rank‐sum test.

### 2.3 Immunohistochemistry

The handling of patient material was performed according to the Göttingen ethics committee vote No. 13/5/17 and according to the Statement of the National Ethics Council on Biobanks for Research, Berlin, Germany and the Declaration of Helsinki. Human melanoma samples were collected only from patients who signed informed consent. Samples were pseudo‐anonymized, and immunohistochemical staining was performed as described previously [20]. Briefly, 5‐μm slides of paraffin‐embedded normal skin, primary melanoma, and metastasis samples were deparaffinated and treated with target retrieval solution (Dako #S1699) for 20 min. After cooling, endogenous peroxidases were blocked with 3% hydrogen peroxide, washed with PBS three times, and stained with primary antibody anti‐PXDN/VPO1 (Merck Millipore, #ABS1675) overnight in a wet chamber at 4 °C. Samples were afterward washed with PBS three times. Secondary antibody (goat‐anti‐mouse Vector #BA‐9200, dilution 1:150; rabbit‐anti‐goat Vector #BA‐5000, dilution 1:150; goat‐anti‐rabbit Vector #BA‐1000, dilution 1:150) was incubated and washed exactly as the primary antibody. Samples were blocked with streptavidin peroxidase (Calbiochem #189733, Burlington, MA, USA). Photographs were taken with an Axio Imager M1 and recorded using the AxioVision software Rel 4.7 (Zeiss, Göttingen, Germany).

### 2.4 CRISPR‐Cas9 knockdown cell line generation

To generate PXDN‐depleted cell lines, we applied CRISPR‐Cas9 technology from Integrated DNA Technologies (IDT). Predesigned guide RNA (Hs.Cas9.PXDN.1.AA) targeting exon 8 of PXDN coding region (5′‐GAAGTACACGGTGTTCCCCG‐3′) was used together with the scaffold Alt‐R^®^ CRISPR‐Cas9 tracrRNA (IDT, #1072532), Cas9 endonuclease Alt‐R^®^ S.p. Cas9 Nuclease V3 (IDT, #1081058) for ribonucleoprotein (RNP) complex formation according to the manufacturer's instructions. For the delivery of the RNP complex, Alt‐R^®^ Cas9 Electroporation Enhancer (IDT, #1075915), SF Cell Line 4D‐Nucleofector™ X Kit S (Lonza, #V4XC‐2032) were used according to the manufacturer's protocols. Program CU‐137 of the 4D‐Nucleofector^®^ was used for electroporation. For control cell lines, non‐targeting crRNA (5′‐CGTTAATCGCGTATAATACG‐3′) from IDT (#1072544) was used for RNP formation. Single‐cell‐derived colonies were expanded and preserved in cryotubes in liquid nitrogen. Primers flanking the guide RNA target site were used for PCR amplification Forward: 5′‐CTTGGTGTGGTTCCCTCATG‐3′, Reverse: 5′‐CGTCTTAGCCTTAACAGGTCC‐3′. PCR products were cleaned up using (PCR clean up kit) followed by Sanger sequencing analysis. Sequence viewing and analysis were done in Snapgene viewer.

### 2.5 Antibodies

Mouse anti‐β‐Actin (Sigma‐Aldrich, #A5541), Rabbit anti‐GAPDH (14C10) (Cell Signaling Technologies, Danvers, MA, USA; #2118), anti‐V5 tag (ThermoFisher, #R960‐25), anti‐His tag (Sigma, #SAB4301134), Rabbit anti‐PXDN/VPO1 (Abclonal, Andover, MA, USA; #A17929), IRDye 680LT Donkey anti‐Mouse (Li‐Core, #926‐68 022), and IRDye 800CW Donkey anti‐Rabbit (Li‐Core, #926–32 213) were used for western blotting. Mouse anti‐SERCA2 (ThermoFisher, #MA3‐919) and Rabbit serum against PXDN (kindly provided by Prof. Miklos Geiszt) were used for immunofluorescent imaging. Mouse anti‐PXDN (2C11) (Santa Cruz Biotechnology, Dallas, TX, USA; #sc‐293 408) was used for PXDN blocking during real‐time killing assay. PE‐conjugated MICA/MICB antibody (Miltenyi Biotech, #130–100‐889; Bergisch Gladbach, Germany), REA Control Antibody (Miltenyi Biotech, #130–113‐438), and Alexa Fluor^®^ 647‐conjugated phospho‐Histone H3 (Ser10) (Cell Signaling Technologies, #3458) were used for flow cytometric analysis.

### 2.6 Western blot analysis

Cells were washed with phosphate‐buffered saline (PBS) followed by trypsinization. Cell pellets were then lysed at 4 °C in RIPA buffer (50 mm Tris/HCl, pH 8.0, 0.5% Na‐deoxycholate, 0.1% sodium dodecyl sulfate (SDS), 120 mm NaCl), supplemented with protease inhibitors (cOmplete™ Protease Inhibitor Cocktail, Roche #11697498001, Mannheim, Germany) and phosphatase inhibitors (PhosSTOP™, Roche # 4906845001). Final concentration of 10 mm NaF, 1 mm phenylmethylsulfonyl fluoride (PMSF), and 1 mm Na‐vanadate were added freshly to the lysis buffer prior to cell lysis. Cell lysates were vortexed and placed on ice for 15 min for complete homogenization. 4× Laemmli Sample Buffer (Bio‐Rad #1610747, Munich, Germany) was added to the lysate followed by denaturation at 95 °C for 10 min. Protein samples were resolved by SDS‐polyacrylamide gel electrophoresis (PAGE) and blotted on the nitrocellulose membrane by using Trans‐Blot Turbo RTA Mini 0.2 μm Nitrocellulose Transfer Kit (Bio‐Rad #1704270). Membranes were blocked in PBS containing either 5% skimmed milk or 5% Bovine Serum Albumin (BSA) (Sigma #A8531), and incubated in PBS containing primary antibody (1:500–1:1000 dilution) and 0.01% sodium azide at 4 °C for 1 h with constant agitation. Membranes were washed three times with tris‐buffered saline (TBS) (150 mm NaCl, 50 mm Tris/Cl, pH 7.5) containing 0.1% Tween 20 (TBST), incubated in fluorescent secondary antibody diluted 1:10 000 in TBST at room temperature for 30 min, and washed again three times with TBST. Imaging and quantification of the blots was performed using an Odyssey infrared imaging system (LI‐COR, Lincoln, NB, USA) and Image Studio™ Lite Software, respectively.

### 2.7 Colony formation assay

10 000 melanoma cells were seeded homogenously into 6‐well plates containing prewarmed culture medium. Cells were cultured at 37 °C in 5% CO_2_ for 14–21 days until visible cell colonies were formed. Cells were washed twice with 1 × PBS followed by fixation with 4% paraformaldehyde (PFA) at room temperature with constant agitation for 15 min. Cells were washed twice with 1 x PBS to remove remaining traces of PFA. Fixed cells were then stained with 1 mL of crystal violet staining solution and were kept at room temperature for 20 min with constant shaking. Stained cells were washed twice with distilled water and air‐dried at room temperature. Representative images were acquired by using the Epson Perfection V850 and processed by using Fiji. For quantification, cells were de‐stained with 800 μL of 40% acetic acid at room temperature for 20 min with constant shaking. De‐stained solutions were diluted 4 times with distilled water prior to absorbance measurement (590 nm) using the Mithras LB 940 plate reader (Berthold Technologies, Bad Wildbad, Germany).

### 2.8 Flow cytometry

Cells were washed with PBS, fixed with 2% paraformaldehyde (PFA, AppliChem, Darmstadt, Germany) diluted in PBS at 37 °C for 10 min and permeabilized with 100% ice‐cold ethanol. Cells were resuspended in PBS containing 4 μg·mL^−1^ propidium iodide (PI, AppliChem) and 50 μg·mL^−1^ RNase A (AppliChem) and incubated for 1 h at 37 °C protected from light. Cell cycle distribution was analyzed using a FACSCanto II flow cytometer (BD Biosciences, San Jose, CA, USA). Individual cell cycle stages were defined by manual gating according to the PI (DNA content) signals. Sample preparation for mitotic cells (p‐H3 positive) and S‐phase cells (EdU positive) for cell cycle analysis was performed as described in a previous study [41]. For quantification of mitotic cells, cells were blocked with PBS containing 1% FBS and stained with p‐H3 antibody (anti‐phosphohistone H3 (Ser10), Alexa Fluor 647 conjugate, Millipore) followed by PI staining and RNAse A treatment for 1 h. The p‐H3‐positive cell population was gated manually. For detecting cell surface antigens, live cells were washed with PBS and stained with anti‐MICA/B antibody or the respective isotype control. Staining was performed in PBS containing 1% FCS and 2 mm EDTA (antibody dilution 1:1000) for 20 min on ice in the dark. Cells were washed twice with staining buffer and resuspended in 400 μL for acquisition. Quantification of marker abundance was conducted by comparison of the mean fluorescence intensities using FlowJo V10.7.2. Results are presented as net mean fluorescence intensity (Net MFI), calculated by MFI subtracted by background MFI measured in isotype controls. For S‐phase cell cycle analysis, Click‐iT^®^ Plus EdU Flow Cytometry Assay Kits (Life technologies, #C10635) was used according to the manufactural instructions.

### 2.9 Immunofluorescence (IF) microscopy

10 mm coverslips (No. 1.5H, Carl‐Roth, #YX02.2) were placed in 6‐well plates and incubated in antibiotic‐free medium for 30 min prior to cell seeding. For fixation, cells were washed twice with PBS and fixed with ice‐cold methanol at room temperature for 3 min. Cells were washed 7 times with PBS after fixation to remove traces of methanol. For IF microscopy, sample preparation was performed as described in previous work [41]. Briefly, cells were blocked in phosphate‐buffered saline (PBS) containing 3% bovine serum albumin (Sigma, #A8531) for 30 min and then incubated in PBS containing primary antibody (diluted at 1:250–1:1000) overnight at 4 °C with constant agitation. Coverslips were washed 3 times with PBS followed by incubation with PBS containing 1 to 10 000 diluted Cy2‐ or Cy3‐conjugated secondary antibodies (Jackson ImmunoResearch, West Grove, PA, USA; #715–225‐150, #711–165‐152) and 1 μg·mL^−1^ of Hoechst 33342 staining solution (Invitrogen™, Waltham, MA, USA; #H3570) at room temperature for 30 min. After 3 washes with PBS, coverslips were mounted on glass microscope slides with Fluoromount G (Invitrogen™, # 00–4958‐02). Images were acquired using 60× oil immersion objectives (numerical aperture: 1.3) on an Olympus IX83 microscope with a scientific CMOS (SCMOS) camera. Acquisition of images using the cellSens software (version 1.16) and the following filter settings: Hoechst 33342: excitation 387/11 HC (Semrock), beam splitter DA/FI/TR/Cy5‐A‐OMF (Semrock), emission 440/40 HC (Semrock); AlexaFluor 488: excitation 485/20 HC (Semrock), beam splitter DA/FI/TR/Cy5‐A‐OMF (Semrock), emission 525/30 HC (Semrock); AlexaFluor 568: 525/30 HC (Semrock), beam splitter DA/FI/TR/Cy5‐A‐OMF (Semrock), and emission 607/36 HC (Semrock).

### 2.10 RNA isolation and RT‐qPCR

The total RNA was isolated by using QIAamp RNA Blood Mini Kit (Qiagen, Hilden, Germany; #52304). 2 μg of total RNA were reverse transcribed using M‐MLV reverse transcriptase (Promega #M1701) and random hexamers (Thermo Fisher Scientific, #N8080127) according to the manufacturer's instructions. 0.5 μL of cDNA was used for RT‐qPCR using the GoTaq^®^ qPCR and RT‐qPCR Systems (Promega, #A6001) and Bio‐Rad CFX96 Real‐Time System. Acquired CT values were calibrated based on the primer efficiency of individual primer sets. Calibrated CT values of the target mRNAs were normalized to the Calibrated CT values of TATA box binding protein (TBP), which was used as the housekeeping gene. Data were quantified using the 2^−ΔΔCT^ method. Gene‐specific primer sets were purchased from Qiagen or Sigma‐Aldrich. Primer sequences for qPCR are listed below.

TBP forward: 5′‐CGGAGAGTTCTGGGATTGT‐3′

TBP reverse: 5′‐GGTTCGTGGCTCTCTTATC‐3′

PXDN forward: 5′‐ATCTCAGCAACAGCACCTC‐3′

PXDN reverse: 5′‐GAGCCGTGATTCAAGTTTCTTTAT‐3′

### 2.11 2D wound healing assay

To assess the *in vitro* migration ability of cells, 100 000 cells/well were seeded into 96‐well plate 24 h prior to wound generation. Cells were washed twice with 1× PBS to remove remaining trace of serum. A single wound was generated in each well by using a 10 μL tip in combination with a vacuum pump. To rule out the impact of cell proliferation, cells were incubated with serum‐free culture medium and recovered in the incubator for 30 min before the acquisition of the first time point (0 h). Bright field images were acquired at indicated time points using 10× objectives on an Olympus IX83 microscope with a scientific CMOS (SCMOS) camera. Acquired images were subjected to ImageJ analysis for quantifying cell‐free area (CFA) of individual images using an ImageJ macro. Percentage of wound closure was quantified by using the following equation: (CFA_t0_ − CFA_t24_)/CFA_t0_ * 100%.

### 2.12 3D spheroid invasion assay

Spheroids were generated as previously described [42]. In short, 5000 cells/well were seeded in 96‐well plates on top of a non‐adhesive layer of 1.5% agar. Following 96 h and the formation of a 3D structure, spheroids were manually harvested and embedded in a collagen I mixture and were allowed to invade for a given period of time. Spheroids were then stained with the Live/Dead Viability/Cytotoxicity Kit (#L3224, Invitrogen) combined with Hoechst 33342 dye (final concentration 1 ng·mL^−1^) and incubated at 37 °C protected from light for 30 min. Images were acquired using 10× objectives on an Olympus IX83 microscope with a scientific CMOS (SCMOS) camera. Acquisition of images was performed using the cellSens software (version 1.16) and the following filter settings: Hoechst 33342: excitation 387/11 HC (Semrock), beam splitter DA/FI/TR/Cy5‐A‐OMF (Semrock), emission 440/40 HC (Semrock); AlexaFluor 488: excitation 485/20 HC (Semrock), beam splitter DA/FI/TR/Cy5‐A‐OMF (Semrock), emission 525/30 HC (Semrock); AlexaFluor 568: excitation 525/30 HC (Semrock), beam splitter DA/FI/TR/Cy5‐A‐OMF (Semrock), emission 607/36 HC (Semrock). Multiple images were taken with the z‐stack mode with the image interval of 1 μm. Spheroid size evaluation was determined using the ImageJ software. Spheroid invasion was measured by subtracting the mask for the core of each spheroid from the total area covered by all the cells of a given spheroid (invasion area [μm^2^] = total area − spheroid core) using ImageJ. Cell death marked by Ethidium Homodimer‐1 (EthD‐1) puncta was quantified by using a self‐developed imagej Macro.

### 2.13 Real‐time NK cell killing assay

Human primary NK cell isolation and the subsequent melanoma killing assay were performed as described previously [7]. Briefly, leucocyte reduction chambers were provided by the local blood bank (University Medical Center Göttingen, Ethics approval 2/3/18) from healthy, non‐smoking donors. Peripheral blood mononuclear cells (PBMCs) were isolated using density gradient centrifugation. Subsequent NK cells isolation was performed by negative bead isolation (Miltenyl Biotec, #130–092‐657). Target cells were loaded with 0.5 μM calcein‐AM (Thermo Fisher Scientific, #C1430) and seeded in a black, clear bottom 96‐well plate. Primary human NK cells were stimulated with Interleukin‐2 (0.05 μg·mL^−1^; Thermo Fisher Scientific GmbH, #15596‐026) 36 h prior to the killing assay. Interleukin‐2‐stimulated primary human NK cells were added to target cells in a NK cell to target cell ratio of 5:1. NK cell‐mediated melanoma killing was measured for 2 h at 37 °C in 5% CO_2_ using a CLARIOstar^®^ plate reader (BMG LABTECH, Ortenberg, Germany). Killing efficiency was evaluated by the decrease in fluorescent signal.

### 2.14 Recombinant PXDN purification

The recombinant human PXDN was provided by the VIB Protein Core facility. The gene coding for human PXDN was cloned inside a pmS‐Hc plasmid for secreted expression from mammalian HEK293F cells. As PXDN contains a N‐terminal His_6_ tag, the secreted protein undergoes an immobilized metal affinity purification using Ni‐sepharose. The medium fraction was dialyzed by tangential filtration on a 30 kDa MWCO filter to diafiltration buffer (50 mm HEPES, 500 mm NaCl, 20 mm imidazole, pH 7.5). The diafiltered medium was loaded on a 6.3 mL Ni^2+^‐Sepharose column and equilibrated with 20 column volumes of diafiltration buffer, the column was eluted with 50 mm HEPES, 20 mm NaCl, 400 mm imidazole, pH 7.5 after an intermediate elution step with 50 mm imidazole. The 400 mm imidazole elution fractions were pooled together for injection on a Superdex200 size exclusion chromatography column to the final PBS formulation buffer. The purity of the protein was estimated higher than 90% on Coomassie gel and has less than 5EU/mg LPS contamination.

### 2.15 Assessment of the oligomeric state of PXDN by mass photometry

The oligomeric state was determined as described (https://www.biorxiv.org/content/10.1101/2025.03.03.641172v1.ful). Briefly, 1 μm PXDN sample was incubated with and without 5 mm dithiothreitol (DTT) in PBS pH 7.6 buffer at room temperature. Protein landing was recorded using a Refeyn OneMP (Refeyn Ltd) MP system by adding 10 μL of a 10× dilution of the protein stock solution (1 μM) into a 10 μL drop of filtered PBS pH 7.4 buffer. Movies (6000 frames, 60 s) were acquired with the AcquireMP (version 2.1.1; Refeyn Ltd) software using default settings. Data were analyzed using default settings on DiscoverMP (version 2.1.1; Refeyn Ltd). Contrast‐to‐mass calibration was performed with MassFerence P1 (Refeyn) using standards of 88, 172, 258, and 344 kDa. The binding and unbinding events were grouped into mass ranges (binning) using GraphPad Prism. Frequency distribution with default setting and a bin width of log_2_(*x*), being *x* the total number of detected particles, was used. Data were represented as number of particles (counts) vs. mass (kDa). Triplicates of each sample were measured.

### 2.16 Ferrous oxidation in xylenol orange (FOX) assay

FOX assay was used to determine the H_2_O_2_ consumption over time by the recombinant purified PXDN as previously described [43, 44]. Briefly, the reaction mixture contained PXDN (1.5 μm) and 200 μm H_2_O_2_ in 100 mm Na‐Phosphate pH7.4, 0.2 m NaCl. At different time points, 10 μL of the reaction mixture were mixed with 490 μL of the FOX reaction mix (100 μm xylenol orange, 250 μm ammonium ferrous sulfate, 100 μm sorbitol and 25 mm H_2_SO_4_) and incubated for 30 min at room temperature in the dark. At the end of the reaction, A560nm was measured in a 96‐well plate reader (SPECTRAmax 340PC, Molecular Devices). The final concentrations of the controls in the reaction mixture are 1.5 μm BSA (Merck) and 1.5 units catalase (Merck). Extra reduction of PXDN with 0.5 μm DTT was also tested.

### 2.17 Lipoperoxidase assay

Lipoperoxidase activity was assessed using the ferrous oxidation–xylenol orange (FOX) assay. The FOX reagent was freshly prepared and contained 100 μm xylenol orange, 250 μm ammonium ferrous (II) sulfate, 100 mm sorbitol, and 25 mm H_2_SO_4_. A 29 mm stock solution of 4‐hydroperoxy‐2‐nonenal (HNE; Cayman Chemical Company – 1 004 413) was prepared in acetone and diluted to a 200 μm working solution in ethanol:PBS (1:7, v/v).

Protein samples included a negative control (1.5 μm BSA), a positive control (0.5 μm human GPX4 (Cayman Chemical Company – 26 906)), and the test sample (0.5 μm PXDN; purified and provided in PBS by the VIB Protein Core Facility). Reactions were initiated by incubating protein samples with 300 μm HNE and 0.5 mm reduced glutathione (GSH) at room temperature. At selected time points, 10 μL aliquots were mixed with 490 μL of FOX reagent and incubated for 30 min in the dark. 200 μL of the mixture was transferred to a 96‐well plate, and absorbance was measured at 560 nm using a SpectraMax 340PC384 plate reader (Molecular Devices).

### 2.18 Recombinant PXDN addition and PXDN inhibition during killing assay

To assess the function of recombinant PXDN in blocking NK cell killing, primary NK cells were incubated with recombinant PXDN (64.5 μg·mL^−1^) at room temperature for 10 min and eventually with the concentration of 20 nm of recombinant PXDN during the killing assay. For PXDN inhibition, PXDN antibody (2C11) (Santa Cruz, sc‐293 408) with the final concentration of 5 μg·mL^−1^ was added to the target melanoma cell suspension prior to cell seeding for the real‐time killing assay.

### 2.19 Transient transfection and plasmids

pcDNA3.1/PXDN‐V5‐His and control plasmid were purified by using Plasmid Maxi Kit (QIAGEN, # 12165) followed by the manufacturer protocol. For transfection, 2 μg of purified plasmid DNA was transfected into melanoma cells (3 million cells per transfection) by using the SF Cell Line 4D‐Nucleofector™ X Kit L (Lonza, # V4XC‐2012). The program CU‐137 of the 4D‐Nucleofector^®^ was used for electroporation.

### 2.20 Modeling and simulation of PXDN‐NK cell receptor complexes

#### 2.20.1 Modeling of complexes

We used AlphaFold 2‐multimer in ColabFold (v. 1.3.0) [45] to model the PXDN homotrimer as well as complexes with putative binding partners. The models were generated in batch mode with 48 recycles and GPU relaxation of the final models. To speed up the computations, individual domains of PXDN were modeled at a time rather than full‐length PXDN. For the PXDN homotrimer we repeated the prediction with AlphaFold 3. The resulting models were analyzed regarding their structural arrangement and their predicted aligned error (PAE), in particular between the individual proteins or domains.

#### 2.20.2 Molecular dynamics simulations

MD simulations were performed as described [46]. Briefly, for selected complexes, we performed molecular dynamics simulations using GROMACS v. 2021.3 [47]. The complexes as modeled by ColabFold were solvated in a box of TIP4P‐D water leaving at least 1.4 nm between the proteins and any box boundary. The DES‐Amber force field [48] was used for the proteins. Sodium and chloride ions were added to neutralize the system and establish an ion concentration of 0.15 mol·l^−1^. To remove initial clashes from the modeling or box packing, steepest descent energy minimizations were run independently on the solvent, the proteins, and finally the whole system. During minimizations, all backbone atoms were kept in place by positional restraints of 500 kJ·mol^−1^·nm^−2^.

For the simulations, the leap‐frog algorithm was used with an integration time step of 2 fs. Nonbonded interactions were treated with a Verlet list cutoff scheme with a 1.0 nm cutoff. Long‐range dispersion correction was applied to energy and pressure. Particle‐mesh Ewald method was used to treat long‐range electrostatic interactions with a gridspacing of 0.12 nm. The LINCS algorithm was used to constrain bonds with hydrogen atoms. A temperature of 298 K was established over 50 ps of simulation under the NVT ensemble applying velocity rescaling with separate heat bath couplings for solvent and solute [49]. Over a further 1000 ns of simulation under the NPT ensemble the pressure was equilibrated at 1 bar using the Berendsen barostat. Positional restraints on the protein were applied to prevent premature complex dissociation during the equilibration steps. Finally, the simulations of the complexes were run without restraints for 500 ns under the NPT ensemble. Coordinates were recorded every 200 ps.

### 2.21 Structure prediction using AlphaFold 3

Protein complex structures were predicted using AlphaFold 3 [50], run locally via a singularity container on a multi‐GPU system. The AlphaFold 3 container (alphafold3.sif) and associated scripts were obtained from the DeepMind GitHub repository. Input sequences were defined in a JSON configuration file specifying the trimeric version of PXDN.

### 2.22 RMSD analysis

To analyze overall motions of the proteins, the root mean square deviation (RMSD) of Cα atoms was calculated using the GROMACS’ rms module. The starting structure was used as a reference. The RMSD was separately calculated for each protein to detect motions within each protein, the complex to assess the complex stability, and the proteins toward each other.

### 2.23 Calculation of binding energies

To calculate the binding energies using the MM‐GB/SA approach, the GROMACS topologies were transformed into the Amber‐format using ParmEd [51]. Then all water molecules and ions were removed from the trajectories. The actual calculations were done using MMPBSA.py [52]. The gas phase energies, that is, van der Waals and electrostatic interactions, were calculated based on the force field. Polar contributions to the solvation free energy were calculated using the OBC^II^ Generalized‐Born implicit solvent model [53] at a salt concentration of 0.15 mol·L^−1^. Nonpolar contributions to the solvation free energy were calculated based on the solvent‐accessible surface area using a surface tension of 0.005 kcal·mol^−1^·Å^−2^. Entropic changes were not considered in the calculation. Finally, individual contributions to the binding energy per residue were calculated using the decomposition scheme implemented in MMPBSA.py.

### 2.24 RNA sequencing analysis

Melanoma cells were seeded at 50% confluency 48 h prior to RNA extraction. Total RNA was isolated at linear growth phase by using QIAamp RNA Blood Mini Kit (Qiagen #52304). RNA samples were submitted to NGS Facility for Integrative Genomics (NIG) for RNA sequencing and downstream analysis identifying differentially expressed genes (DEGs).

## 3 Results

### 3.1 Peroxidasin is upregulated in melanoma

**Fig. 1:**
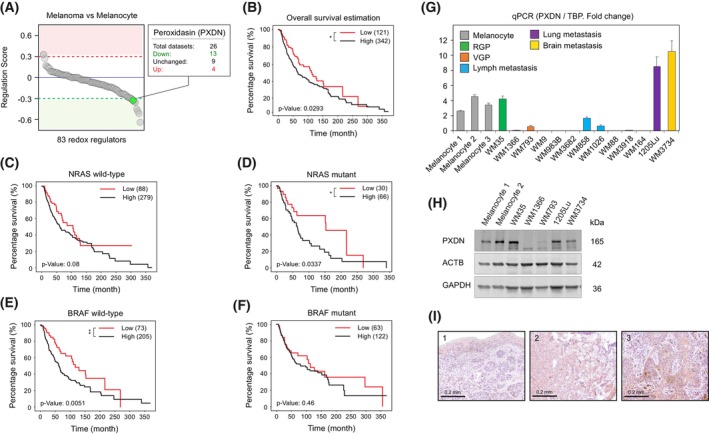
Identification of *PXDN* as an oncogene in metastatic melanoma cells. (A) Bioinformatic analysis indicating Peroxidasin (*PXDN*; green) as a redox regulator downregulated during melanocyte malignant transformation. Twenty‐six microarray datasets acquired from Gene Expression Omnibus (GEO) database were used to calculate the regulation score of 83 selected redox‐regulating proteins during melanocyte malignant transformation. Numbers of datasets showing down‐ (green), up‐ (red) regulation, or no significant changes in *PXDN* expression during malignant transformation are indicated. (B–F) Kaplan–Meier survival estimation based on TCGA melanoma patient data comparing high‐ (black) or low‐*PXDN* (red) expression under different genetic backgrounds. (B) Overall survival, (C) wild‐type NRAS, (D) mutant NRAS, (E) wild‐type BRAF, (F) mutant BRAF. *P*‐values calculated using a log‐rank test. *P < 0.05, **P < 0.01. (G) RT‐qPCR analysis of *PXDN* mRNA expression in healthy melanocytes (gray) and melanoma cell lines derived from patients at different disease stages. Radial growth phase (RGP): green; vertical growth phase (VGP): orange; lymph metastasis: blue; lung metastasis: purple; brain metastasis: yellow. Relative fold change of PXDN normalized to TATA‐Box Binding Protein (TBP). Error bars indicate mean values ± SEM; *n* = 4 independent experiments. (H) Western blot analysis of PXDN protein abundance in healthy melanocytes and melanoma cell lines at different disease stages. Beta‐actin (ACTB) and glyceraldehyde‐3‐phosphate dehydrogenase (GAPDH) used as loading control; *n* = 3 independent experiments. (I) Representative PXDN immunohistochemistry images (from three independent experiments, *n* = 3) in tissue samples from primary melanoma (panel 1) and metastatic melanoma (panels 2 and 3). Scale bar = 0.2 mm.

To identify redox regulators relevant to melanoma, we curated a list of genes encoding redox‐active proteins based on Gene Ontology (GO) annotations and compared their expression between healthy melanocytes and malignant melanoma cells. Using 16 microarray datasets from the Gene Expression Omnibus (GEO), we generated 29 differential gene expression (DGE) profiles to compute a regulation score for each gene (Table S1). Applying an empirical cutoff (upregulation >0.3; downregulation <−0.3) to reduce preliminary hits, we identified nine significantly dysregulated genes: *GSTP1, PXDN, FAM213A, APOE, FAM213B, SEPW1, GPX7*, *and CYGB* (Fig. 1A, Table S1). Among these, peroxidasin (*PXDN*) stood out due to its known feature in being upregulated in multiple cancer entities compared to their healthy counterparts. A recent study by Li et al. corroborated our findings by demonstrating that among skin cell melanoma (SKCM), metastatic melanoma exhibits higher *PXDN* expression compared to primary melanoma [36].

Based on these findings, and given that PXDN is a secreted protein capable of influencing cell–cell interactions and potentially contributing to cancer cell immune evasion, we focused on elucidating its role in melanoma progression and evaluating its therapeutic potential.

### 3.2 High PXDN levels correlate with poor prognosis and melanoma aggressiveness

To assess the impact of PXDN on melanoma patient survival, we classified patients from TCGA datasets into high and low *PXDN* expression groups and performed Kaplan–Meier survival analyses. Patients with low *PXDN* expression showed significantly better overall survival (Fig. 1B). This negative correlation (high expression = low survival) was even stronger in patients with *NRAS* mutations (Fig. 1C,D) and in *BRAF* wild‐type (WT) patients (Fig. 1E), while the difference was less prominent and not significant in *BRAF*‐mutant cases (Fig. 1F). Stage‐specific analysis further revealed that the negative correlation between *PXDN* expression and survival was more evident in early‐stage (0–II) than late‐stage (III–IV) melanoma, particularly in *BRAF* WT patients (Fig. S1a,b). We next examined whether *PXDN* expression is affected by common melanoma driver mutations and found no significant differences in *PXDN* mRNA levels between WT and mutant *BRAF* or *NRAS* patients (Fig. S1c,d), nor between patients with remission versus progression outcomes (Fig. S1e).

To validate these findings experimentally, we performed RT‐qPCR on healthy melanocytes and melanoma cell lines representing various disease stages. *PXDN* expression was highest in aggressive metastatic lines from lung (1205Lu) and brain (WM3734), showing a ~2‐fold increase over melanocytes (Fig. 1G). Primary melanoma cells (WM35) under radial growth phase (RGP) showed no change, while other less aggressive lines were downregulated. Immunoblotting confirmed these expression differences across progression stages (Fig. 1H).

To investigate PXDN in clinical samples, we performed immunohistochemistry (IHC) staining on patient‐derived melanoma tissues and observed elevated PXDN abundance at metastatic sites (Fig. 1I).

Together, these findings indicate that high *PXDN* expression is associated with reduced survival, especially in *BRAF* WT and early‐stage patients and correlates with melanoma aggressiveness and metastatic progression.

### 3.3 PXDN depletion impairs melanoma cell growth and disrupts cell cycle progression

**Fig. 2:**
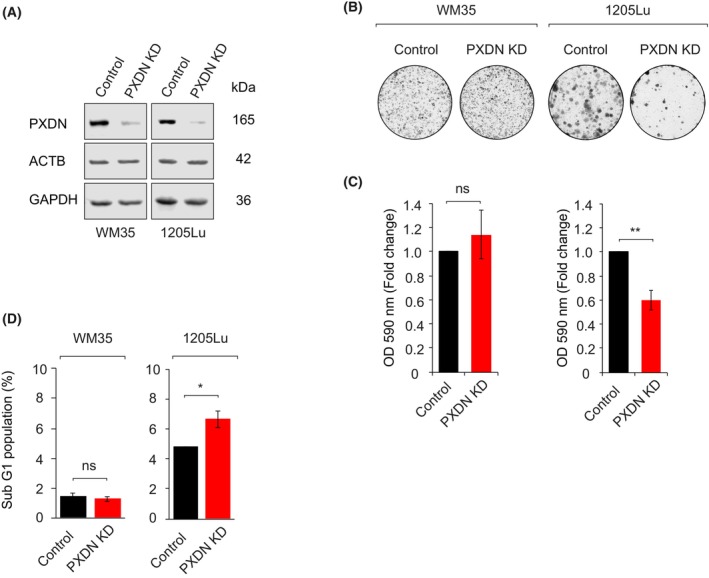
PXDN controls melanoma colony formation and cell cycle progression. (A) Western blot analysis comparing the abundance of endogenous PXDN between control and PXDN knockdown (KD) in WM35 and 1205Lu cell lines; *n* = 3 independent experiments. (B, C) Colony formation assay comparisons between control and PXDN KD in WM35 and 1205Lu cell lines. Representative images (B) and quantification; *n* = 3 independent experiments (C) of crystal violet absorbance (595 nm); *n* = 3 independent experiments. (D) Quantification of cells in sub‐G1 phase. DNA content was stained with Hoechst 33342. Cell cycle profiles acquired by using flow cytometry. Sub‐G1 populations gated empirically and quantified by using FACSCanto II flow cytometer (BD Biosciences). Error bars indicate mean values ± SEM; *n* = 4 independent experiments. *P*‐values were calculated using an unpaired Student *t*‐test. ns = no significant difference, *P < 0.05, **P < 0.01.

To further examine the importance of PXDN in melanoma aggressiveness, we selected WM35 (RGP, low PXDN) and 1205Lu (lung metastasis, high PXDN) cell lines for further analysis (Fig. 1G,H). *PXDN* was depleted using CRISPR‐Cas9, achieving >70% knockdown in both lines (Fig. 2A). Sanger sequencing revealed that a single nucleotide deletion results in *PXDN* depletion by introducing premature stop codons (Fig. S2a).

Colony formation assays revealed a significant reduction in colony number and cell viability (~40%) in PXDN‐depleted (PXDN KD) 1205Lu cells, but no change in WM35 (Fig. 2B,C). To evaluate cell cycle effects, we performed flow cytometry combined with EdU incorporation. *PXDN* depletion decreased S‐phase cells and increased G2/M phase cells in WM35 (Fig. S2b,c), while EdU mean fluorescence intensity (MFI) dropped by ~25% in both lines, indicating reduced DNA replication (Fig. S2d–f).

We also determined mitotic cells via phospho‐histone H3 (p‐H3) staining and found a 20–25% reduction in M phase cells in both lines upon *PXDN* knockdown (Fig. S2g–i). To assess apoptosis, we quantified the sub‐G1 population and observed a 40% increase in 1205Lu PXDN KD cells, but no significant change in WM35 (Fig. 2D), consistent with the differential colony formation phenotype (Fig. 2B,C).

Overall, we found that *PXDN* depletion disrupts S‐phase progression, G2/M transition, and mitosis, leading to genomic instability and increased cell death, effects more pronounced in metastatic (1205Lu) than in primary (WM35) melanoma cells. These findings suggested that metastatic melanoma cells are more reliant on PXDN for maintaining cell cycle control and survival.

### 3.4 PXDN promotes melanoma invasion and survival

Previous studies suggested that PXDN is involved in cell migration and invasion [33, 34, 35]. To assess the role of PXDN in melanoma aggressiveness, we initially performed a 2D wound healing assay using three melanoma cell lines representing different disease stages: WM35 (RGP), WM793 (VGP), and 1205Lu (metastatic). Migration rates correlated with disease stage, with WM35 migrating the slowest and 1205Lu the fastest (Fig. S3a,b) However, PXDN depletion did not significantly alter 2D migration in either WM35 or 1205Lu (Fig. S3c–f).

**Fig. 3:**
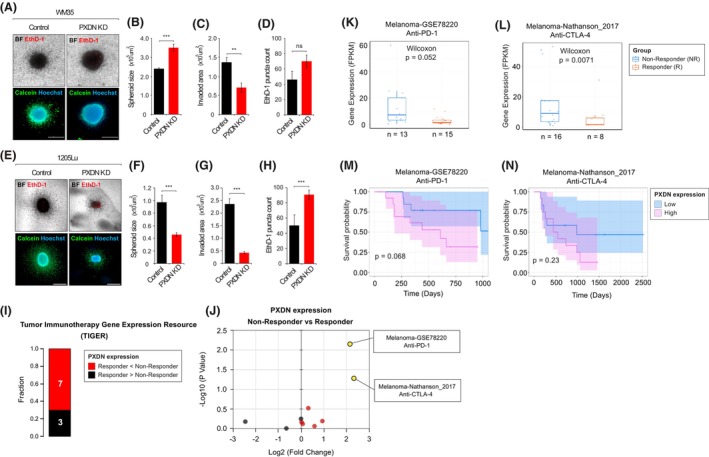
PXDN controls melanoma invasion and responsiveness to immunotherapy. (A) Representative images of WM35 control (Left) and WM35 PXDN knockdown (KD) (Right) melanoma spheroids after 48 h invasion in a collagen I‐based solution. Images of 5 control spheroids and 5 PXDN KD spheroids were acquired from 2 independent experiments. BF, bright field; EthD‐1, Ethidium Homodimer‐1. Scale bar = 500 μm. (B–D) Quantification of spheroid size (B), invasion area (C) and cell death puncta number (D) in WM35 control (black) and WM35 PXDN KD (red) spheroids. (E) Representative images of 1205Lu control (Left) and 1205Lu PXDN KD (Right) melanoma spheroids after 24 h invasion in a collagen I‐based solution. Images of 31 control spheroids and 53 PXDN KD spheroids were acquired from 2 independent experiments. Scale bar = 500 μm. BF, bright field; EthD‐1, ethidium homodimer‐1. (F–H) Quantification of spheroid size (F), invasion area (G) and cell death puncta number (H) in 1205Lu control (black) and 1205Lu PXDN KD (red) spheroids. (A–D) WM35 control spheroids (*n* = 5 from 2 independent experiments), WM35 PXDN KD spheroids (*n* = 5 from 2 independent experiments). (E–H) 1205Lu control (*n* = 31 from 2 independent experiments), 1205Lu PXDN KD (*n* = 53 from 2 independent experiments). Error bars indicate mean values ± SEM. *P*‐values calculated using unpaired Student *t*‐test. ns, no significant difference, **P < 0.01, ***P < 0.001. (I, J) Patient data on *PXDN* expression and responsiveness to immunotherapy were obtained from the Tumor Immunotherapy Gene Expression Resource (TIGER; http://tiger.canceromics.org/). Ten datasets from independent studies classifying patients as responders (R) or non‐responders (NR) to anti‐PD‐1 and/or anti‐CTLA‐4 immunotherapies were analyzed. (I) Bar plot showing the fraction of studies indicating pro‐ (Black: 3 datasets) and anti‐immunotherapy (Red: 7 datasets) effects of high *PDXN* expression. (J) Volcano plot illustrating the Log_2_ fold change and −Log(*P*‐value) comparing *PXDN* expression between responders and non‐responders in datasets from panel I. Two datasets showing significantly high *PXDN* expressions in non‐responders (Melanoma‐GSE78220 and Melanoma‐Nathanson_2017) are marked in yellow. (K, L) Box plots adapted from TIGER indicating *PXDN* expressions among responders (red) and non‐responders (blue) to anti‐PD‐1 (K: Melanoma‐GSE78220) and anti‐CTLA‐4 (L: Melanoma‐Nathanson_2017) immunotherapies. *n* indicates patient number in indicated groups. *P*‐values calculated by Wilcoxon rank‐sum test. (M, N) Kaplan–Meier survival estimation plot adapted from TIGER. Comparison between melanoma patients with high (violet) or low *PXDN* (blue) from different datasets (M: Melanoma‐GSE78220; N: Melanoma‐Nathanson_2017). *P*‐values calculated using univariate Cox regression analysis.

To better mimic the *in vivo* tumoral environment [54], we employed a 3D collagen‐embedded spheroid invasion assay. *PXDN* depletion led to an increased spheroid size in WM35 and a marked decrease in 1205Lu (Fig. 3A,B,E,F). Invasion was reduced in both cell lines upon *PXDN* knockdown, with a more pronounced effect in 1205Lu (Fig. 3C–G). Additionally, *PXDN* depletion led to increased cell death, which was assessed by ethidium homodimer‐1 (EhD‐1) staining. The results again revealed a stronger effect of PXDN depletion‐mediated cell death in 1205Lu (Fig. 3D–H).

Together, these results highlight PXDN as a key mediator of melanoma cell invasion and survival, particularly in metastatic cells, aligning with clinical data linking high *PXDN* expression to poor patient prognosis.

### 3.5 PXDN regulates melanoma susceptibility to NK cell‐mediated cytotoxicity

Mitochondrial metabolism and redox signaling influence cancer cell sensitivity to immune responses, including NK cell activity [55, 56, 57, 58]. Our prior work showed that melanoma cell oxygen consumption rates (OCR) correlate with primary NK cell‐mediated killing (*pNKmK*) [7]. Given that we also discovered that upon *PXDN* depletion, both WM35 and 1205Lu showed decreased OCR (data not shown), we further investigated the role of PXDN in modulating melanoma susceptibility to immunotherapy.

Analysis of Tumor Immunotherapy Gene Expression Resource (TIGER) datasets revealed higher *PXDN* expression in non‐responders (NR) vs. responders (R) in 7 of 10 immunotherapy cohorts (Fig. 3I), with significant differences in anti‐PD‐1 (GSE78220) and anti‐CTLA‐4 (Nathanson_2017) datasets (Fig. 3J). In both datasets, non‐responders had significantly elevated PXDN levels (Fig. 3K,L) and poorer survival outcomes (Fig. 3M,N), suggesting PXDN may impair immunotherapy response.

**Fig. 4:**
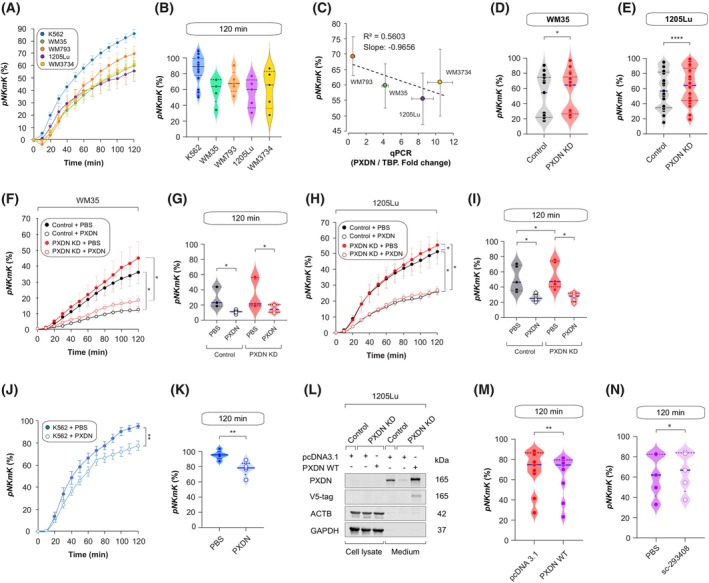
PXDN controls NK cell‐mediated melanoma killing. (A) Kinetics of primary NK cell‐mediated killing (*pNKmK*) of indicated cell lines. (B) Violin plot showing *pNKmK* distribution after 2 h of the killing assay. (A, B) K562 (*n* = 25), WM35 (*n* = 5), WM793 (*n* = 5), 1205Lu (*n* = 5), WM3734 (*n* = 5). (C) Correlation between *pNKmK* at 2 h and relative *PXDN* mRNA expression (normalized to TBP) in indicated melanoma cell lines. RT‐qPCR data were acquired from 4 independent experiments as indicated in Fig. 1G. (D, E) Violin plot showing *pNKmK* at 2 h of the killing assay. (D) WM35 control (black) and WM35 PXDN KD (red) cells (*n* = 18). (E) 1205Lu control (black) and 1205Lu PXDN KD (red) cells (*n* = 21). *P*‐values calculated using paired Student *t*‐test. *P < 0.05, ****P < 0.0001. (F–K) Kinetics of *pNKmK* and violin plot showing *pNKmK* at 2 h of the killing assay with or without recombinant PXDN. (F, G) WM35 control (black) and WM35 PXDN KD (red) (*n* = 5). *P*‐values calculated using paired Student t‐test. *P < 0.05. (H, I) 1205Lu control (black) and PXDN KD (red) cells (*n* = 5). *P*‐values calculated using paired Student t‐test. *P < 0.05. (J, K) K562 (*n* = 5). Recombinant PXDN (20 nm) added during real‐time killing assay. PBS used as control. Median value of *pNKmK* (blue line) and 95% percentiles (dash lines) marked in violin plots. *P*‐values calculated using paired Student t‐test. **P < 0.01. (L–N) Rescue experiment of *pNKmK*. Cells transfected with indicated plasmids for 24 h followed by real‐time killing assay. (L) Representative images of western blot analysis comparing the abundance of PXDN in indicated cells 24 h post‐transfection of indicated plasmids (pcDNA3.1: vehicle control, PXDN WT: pcDNA3.1/PXDN‐V5‐His). *n* = 3 independent experiments. (M) Violin plot showing *pNKmK* of 1205Lu PXDN KD cells transfected with indicated plasmids at 2 h of the killing assay (*n* = 9). *P*‐values calculated by using paired Student t‐test. **P < 0.01. (N) Violin plot showing *pNKmK* of 1205Lu cells treated with indicated antibody at 2 h of the killing assay (*n* = 5). *P*‐values calculated by using paired Student t‐test. *P < 0.05. In all plots, Error bars indicate mean values ± SEM. Median values in violin plots are indicated with solid blue lines. Dash lines in violin plots mark the 5–95% confidence interval.

We next assessed *pNKmK* across melanoma cell lines with varying PXDN levels over a 2 h real‐time killing assay. Susceptibility varied by cell line and disease stage (Fig. 4A,B). Notably, WM793 (VGP) showed ~70% cell death, while its metastatic derivative 1205Lu showed ~55%, indicating acquired resistance to *pNKmK* during progression. PXDN was significantly higher in 1205Lu, as confirmed by immunofluorescence (Fig. S4a) and earlier data (see Fig. 1G,H). Correlation analysis revealed a strong inverse relationship between *PXDN* expression and *pNKmK* efficiency (slope: −0.9656) (Fig. 4C).

To directly test PXDN's role, we evaluated NK susceptibility in PXDN KD vs. control cells. *PXDN* depletion increased *pNKmK* in both WM35 (Fig. 4D) and 1205Lu (Fig. 4E).

Elevated expression of NK cell‐activating antigens such as *MICA* and *MICB* (collectively referred to as MICA/B), which are potent ligands for the NKG2D receptor on NK cells, has been shown to enhance melanoma susceptibility to *pNKmK* [7, 59]. Consistent with this, flow cytometric analysis revealed an approximately 2‐fold increase in MICA/B abundance in PXDN KD WM35 cells compared to control (Fig. S4b). However, no significant difference in MICA/B levels was detected between control and PXDN KD in 1205Lu cells (Fig. S4c). These findings suggest that while *PXDN* depletion can upregulate NKG2D ligands in certain melanoma cell lines, the enhanced sensitivity to pNKmK observed upon *PXDN* knockdown cannot be solely attributed to changes in MICA/B expression.

To further explore this, we performed RNA‐seq on PXDN KD and control cells in both lines. Aside from *PXDN* itself (Fig. S4d), no common differentially expressed genes were found, suggesting that PXDN modulates NK susceptibility independently of specific intracellular signaling cascades.

### 3.6 Extracellular PXDN inhibits NK cell‐mediated cytotoxicity

PXDN is a secreted enzyme that may modulate the tumor microenvironment and suppress antitumor immune responses. To confirm PXDN secretion in melanoma, we performed trichloroacetic acid (TCA)‐mediated protein precipitation, which verified the presence of PXDN in the extracellular milieu of melanoma cell lines (Fig. S5a). Importantly, both intracellular and extracellular PXDN levels negatively correlated with primary NK cell‐mediated killing (*pNKmK*), suggesting a potential role for PXDN in immune evasion (Fig. S5b,c).

To directly investigate the effect of extracellular PXDN on NK cell cytotoxicity, we purified full‐length recombinant PXDN. SDS/PAGE and mass photometry confirmed that the purified protein existed as both monomers and disulfide‐linked trimers, which could be reduced to monomers with dithiothreitol (DTT) (Fig. S5d). Spectral analysis revealed only a minor presence of heme, indicated by a weak shoulder at 410 nm (Fig. S5e) [26]. Functional assays showed that the recombinant PXDN had weak peroxidase activity—<20% of catalase control in hydrogen peroxide decomposition—and limited lipoperoxidase activity when compared to the Gpx4 control (Fig. S5f,g), indicating it was largely peroxidase‐inactive.

To assess whether recombinant PXDN is taken up by melanoma cells, we incubated cells with increasing concentrations of the protein. This led to a dose‐dependent increase in extracellular PXDN without altering intracellular levels, indicating that the protein remains extracellular and is suitable for studying non‐cell‐autonomous effects (Fig. S5h).

We then tested whether extracellular PXDN influences *pNKmK*. Addition of 20 nm recombinant PXDN significantly suppressed NK cell‐mediated killing in both WM35 and 1205Lu melanoma cells. In WM35, *pNKmK* was reduced by 53% in control cells and by 63% in PXDN knockdown (KD) cells. In 1205Lu, PXDN reduced *pNKmK* by 43% in controls and by 50% in PXDN KD cells (Fig. 4F–I). This inhibitory effect extended to K562 leukemia cells, which are highly sensitive to NK cell killing, showing a ~ 20% reduction in *pNKmK*, indicating that PXDN acts directly on NK cells in a tumor‐type‐independent manner (Fig. 4J,K).

To further validate the immunosuppressive role of PXDN, we performed rescue experiments in 1205Lu PXDN KD cells. Transient re‐expression of *PXDN* restored resistance to NK cell killing (Fig. 4L,M), despite minimal recovery of intracellular PXDN (Fig. 4L), suggesting that the restored resistance was mediated by secreted PXDN. Finally, treatment with an anti‐PXDN neutralizing antibody increased *pNKmK*, confirming the functional role of extracellular PXDN in suppressing NK cell activity (Fig. 4N).

In conclusion, these results demonstrate that extracellular PXDN inhibits NK cell‐mediated cytotoxicity in a peroxidase‐independent manner, acting directly on immune effector cells to promote melanoma immune evasion.

### 3.7 PXDN exhibits a trimeric structure with disulfide‐linked peroxidase domains

To determine which domain of PXDN could potentially interact with NK cell receptor(s) or melanoma ligands, we determined the trimeric structure of PXDN [60] using AlphaFold2‐multimer as implemented in ColabFold. Because the prediction of the trimeric structure exceeded resource limitations due to the substantial number of residues involved (3 times 1479 residues), we adopted a stepwise approach.

**Fig. 5:**
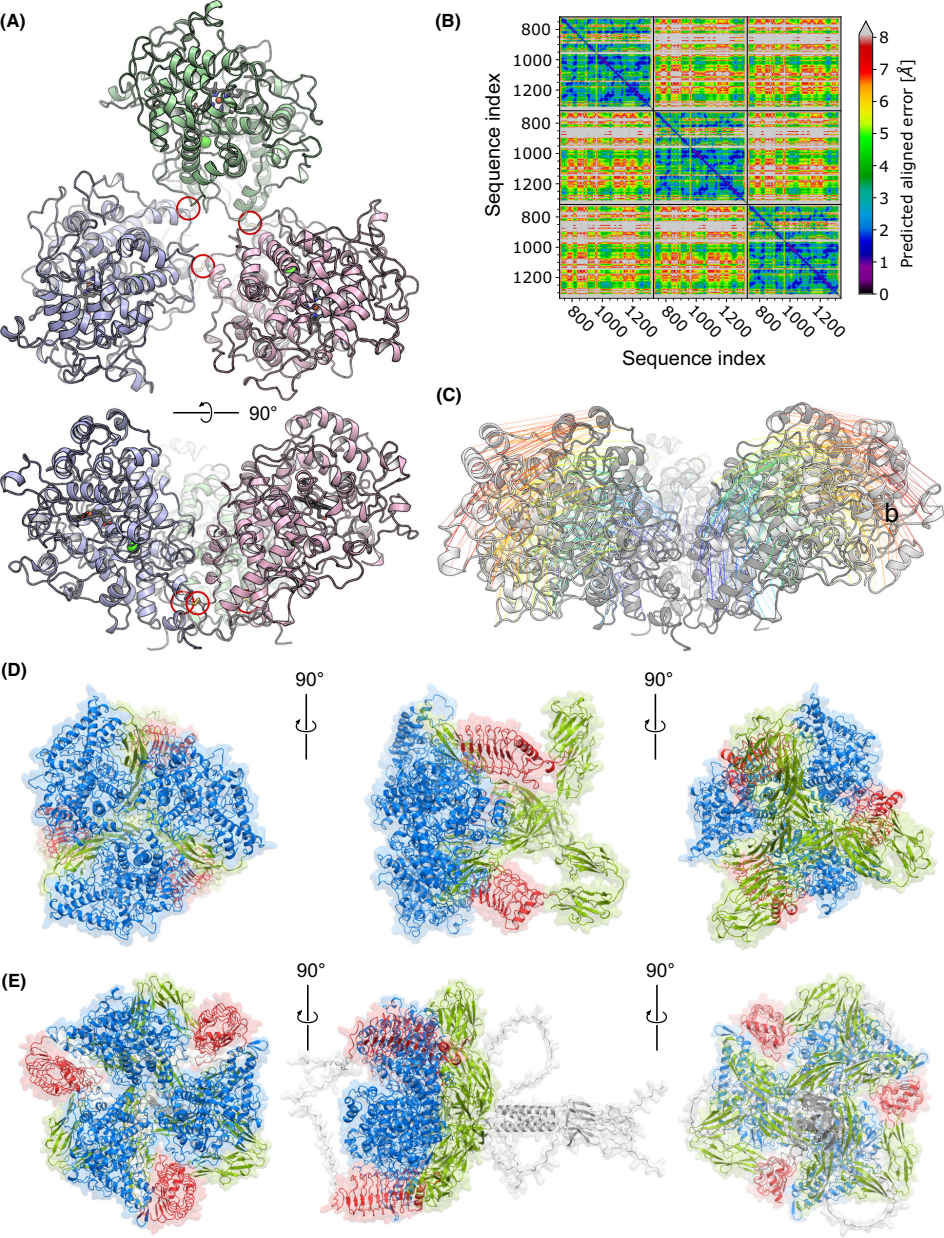
Modeling studies on PXDN. (A) Model of the homo‐trimeric catalytic domain of PXDN. The domains are rotationally symmetric and stabilized by interdomain disulfide bonds (red circles). (B) Predicted aligned error of the homo‐trimeric PXDN model. Black lines in the plot indicate monomer boundaries. (C) Comparison of the trimeric peroxidase domain models of PXDN. The light structure was modeled after a SAXS experiment, while the dark model was generated by AlphaFold2‐multimer. Monomer structures are nearly identical (RMSD 0.7 Å); the quaternary structures differ, with the AlphaFold model monomers rotated inward. (D) Trimeric model of full‐length PXDN, resembling a three ‐blade marine propeller. Peroxidase domains (blue) are connected by disulfide bonds. Four Ig‐domains (green) link the LRR domain (red) to the peroxidase domains. Three distinct orientations are shown. All visualizations of protein structures were generated using PyMOL v.3.1.

First, we focused on the structural model of the three peroxidase domains, including the flanking cysteine regions (3 times 619 residues from ANLS…RGRR) (Fig. 5A). This resulted in a highly reliable disulfide‐linked structure with an average pLDDT value of 91.67%. The pairwise error plot further revealed clear interactions between the three monomers (Fig. 5B). Subsequently, we incorporated three heme groups and Ca^2+^ ions into the structural model using the PDB structure of YAK lactoperoxidase (PDB ID: 7DE5) [61]. AlphaFold 2 accurately left space to accommodate these nonbonded molecules. Notably, our structural prediction of the trimeric conformation showed differences from the triangular arrangement observed through small‐angle X‐ray scattering (SAXS), indicating a planar conformation of the three domains (Fig. 5C). Furthermore, the AlphaFold 2 model suggested dynamic flexibility at the central core of the trimer.

Next, we determined the full‐length structure of the monomer by removing flexible N‐terminal signal peptide and C‐terminal residues, using one copy of 1247 residues (TSIL…VDLR) and two copies of 619 residues (ANLS…RGRR). This resulted in a structure with an average pLDDT score of 85.2% and with a pairwise error plot revealing clear interactions. Aligning the TSIL.VDLR copy which contains the four Ig‐domains and the leucine‐rich repeat (LRR) domain with the two additional peroxidase disulfide‐linked domains resulted in a structure with a three‐fold axis of rotational symmetry resembling ‘a three‐blade marine propeller’ (Fig. 5D). Our model indicated that the N‐terminal LRR domains are connected to the peroxidase domains via four Ig‐domains (Fig. S6). This arrangement positions the LRR domain in close proximity to the fourth Ig‐domain of the monomer, forming a 240‐degree angle. Consequently, the four Ig‐domains bend, creating a structural resemblance to a single blade of a propeller. Our stepwise approach successfully elucidated the trimeric structure of PXDN, highlighting its dynamic flexibility. This unique architecture provides novel insights into potential PXDN–protein interactions and structural stability, which may be critical for PXDN function.

However, caution is warranted in interpreting the presented trimeric structure of PXDN. Existing structural models, while informative, may not fully capture the conformational dynamics or domain flexibility of the full‐length protein. To gain further insight, we generated a structural prediction using AlphaFold 3, employing the complete amino acid sequence of PXDN (residues MAKR…EEKP). The resulting model (Fig. 5E), color‐coded by domain, displays a striking architecture reminiscent of a “bouquet of flowers,” with a pLDDT confidence score of 70.14%.

In this new prediction, the N‐terminal leucine‐rich repeat (LRR) domains form prominent, outwardly projecting “arms” that appear more extended and solvent‐exposed compared to previous models. This spatial arrangement suggests that these LRR domains are structurally positioned for potential interactions with extracellular receptors or immune effector molecules. Their high solvent accessibility and conformational reach make them strong candidates for mediating protein–protein interactions, particularly in the context of immune modulation, such as engagement with NK cell surface receptors. These observations support the hypothesis that PXDN's immune‐regulatory function may be mediated, at least in part, by its extracellular LRR domains.

### 3.8 Molecular dynamics simulations indicate LRR domain‐NKG2D interactions

**Fig. 6:**
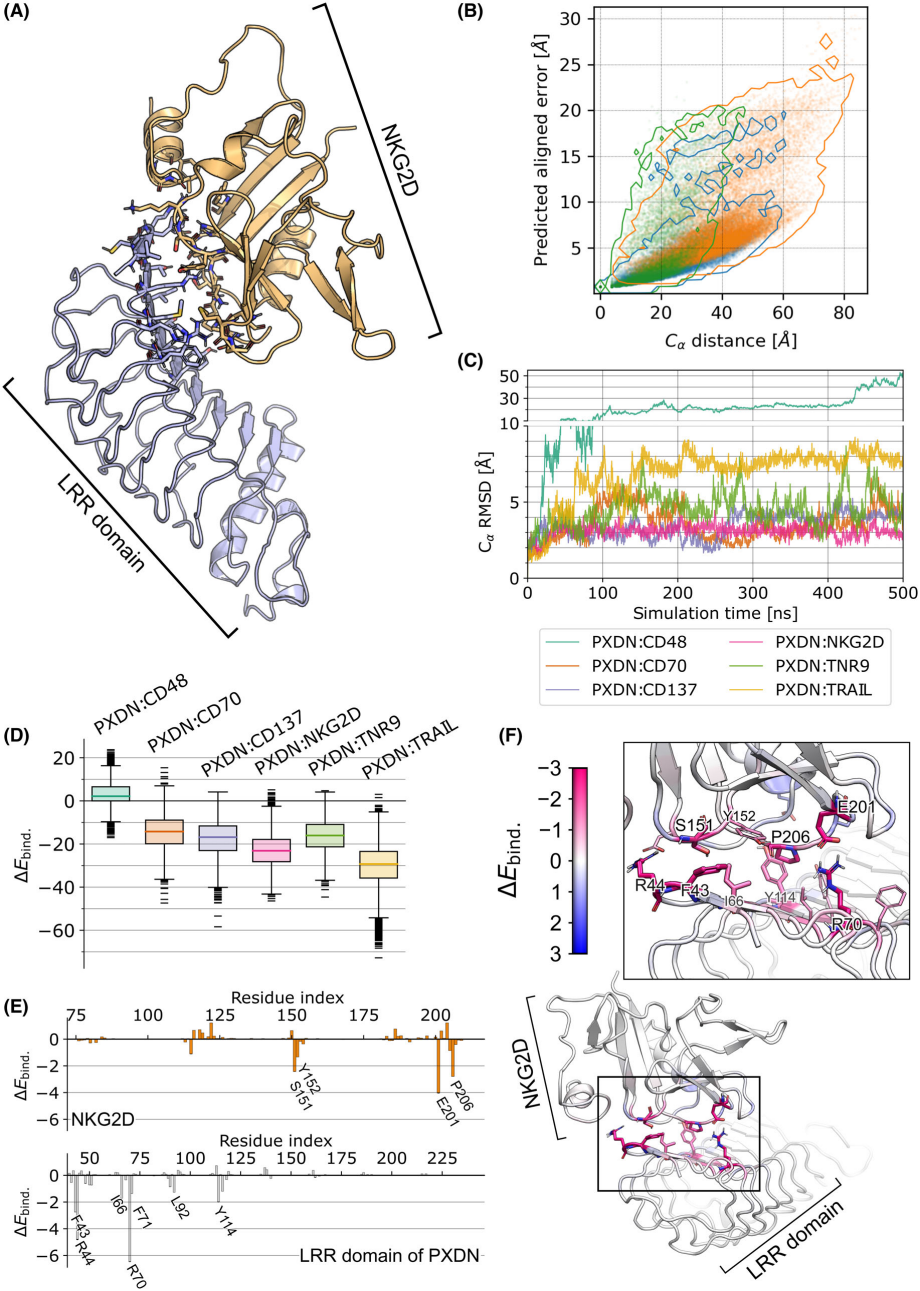
Analysis and simulations on PXDN:NK cell and melanoma receptor complexes. (A) AlphaFold2 model of the LRR domain of PXDN and NKG2D, illustrating a putative complex prediction. (B) Predicted aligned error (PAE) plot for the LRR:NKG2D complex, showing residue‐residue distance. Internal PAE of the LRR domain (blue), internal PAE of the NKG2D domain (green), and interprotein PAE (orange) indicate similar distributions with multiple high‐confidence interprotein contacts. (C) RMSD analysis of the LRR domain of PXDN with NK cell (NKG2D, TNR9) and melanoma (CD70) receptors. (D) Binding energy calculation using the MM‐GB/SA approach. The boxes extend from the first to third quartile (interquartile range, IQR), with the bold line indicating the distribution median. The whiskers extend up to 1.5 IQR beyond the first and third quartile. Data outside that range are shown as dashes. (E) Individual contributions of each residue to the dimerization in the PXDN‐NKG2D model, with key residues labeled. (F) Structural model of the PXDN‐NKG2D interface, with residues colored by their binding energy contributions. Magenta indicates high contribution, while blue indicates detrimental effects. All visualizations of protein structures were generated using PyMOL v.3.1.

To identify potential interaction partners of PXDN on NK‐ and melanoma cells, we used AlphaFold 2‐multimer to model possible complexes of PXDN with NK cell receptors. Previous research indicated a crucial role of the LRR domain in PXDN's high‐affinity binding capacity [62]. Given this valuable insight, we focused on generating complex models involving the LRR domain of PXDN and the extracellular domains of 20 receptors and ligands expressed on NK and melanoma cells, respectively (Fig. 6A, Table 1) [10]. We evaluated their predicted aligned error (PAE) (Fig. 6B), an internal measure of AlphaFold 2 that indicates the uncertainty in the relative positioning of residues. From the 20 complex models initially generated, we retained six with the lowest PAE values (<2.5 Å): PXDN:CD48, PXDN:CD70, PXDN:CD137, PXDN:NKG2D, PXDN:TNR9, and PXDN:TRAIL‐R2. To assess the stability of these complexes in solution, we performed 500 ns unbiased molecular dynamics (MD) simulations and calculated RMSD values. PXDN:CD48 showed complete dissociation (Fig. 6C, Fig. S7a). PXDN:CD70 and PXDN:TRAIL‐R2 remained bound but had high RMSD values, indicating unstable protein–protein interaction (Fig. S7b,f). PXDN:CD137 and PXDN:TNR9 also had high RMSD values due to intrinsic disorder, but their protein–protein interfaces remained stable (Fig. S7c,e). PXDN:NKG2D displayed the lowest RMSD values, maintaining the AlphaFold‐predicted structure throughout the simulation (Fig. 6C, Fig. S7d).

**Table 1:** Overview of the AlphaFold 2‐multimer models on the LRR domain in complex with melanoma and NK cell receptors.

Complex	Stochiometry	Uniprot IDs	Seq. range	Seq. length	PAE (min)	No. contacts
PXDN‐LRR: CD16	1: 1	Q92626 O75015	40–245 22–106	1479 233	6.97	17
PXDN‐LRR: CD27	1: 1	Q92626 P26842	40–245 20–191	1479 260	6.62	19
PXDN‐LRR: CD48^(MD)^	1: 1	Q92626 P09326	40–245 1–243	1479 243	1.09	23
PXDN‐LRR: CD70 ^(MD)^	1: 1	Q92626 P32970	40–245 39–193	1479 193	2.17	25
PXDN‐LRR: CD137 ^(MD)^	1: 1	Q92626 Q07011	40–245 24–186	1479 255	1.10	25
PXDN‐LRR: CD155	1: 1	Q92626 P15151	40–245 21–343	1479 417	9.23	21
PXDN‐LRR: CD226	1: 1	Q92626 Q15762	40–245 19–254	1479 336	13.61	23
PXDN‐LRR: CD244	1: 1	Q92626 Q9BZW8	40–245 22–229	1479 370	8.45	23
PXDN‐LRR: FCG3A	1: 1	Q92626 P08637	40–245 17–208	1479 254	7.61	17
PXDN‐LRR: MICA	1: 1	Q92626 Q29983	40–245 24–307	1479 383	3.46	32
PXDN‐LRR: KI3L1	1: 1	Q92626 P43629	40–245 28–317	1479 444	11.52	33
PXDN‐LRR‐Ig: MICA	1: 1	Q92626 Q29983	27–619 24–307	1479 383	12.08	21
PXDN‐LRR: MICB	1: 1	Q92626 Q29980	40–245 23–309	1479 383	2.56	32
PXDN‐LRR: NCTR3	1: 1	Q92626 O14931	40–245 19–135	1479 201	2.73	19
PXDN‐LRR: NKG2A	1: 1	Q92626 P26715	40–245 94–233	1479 233	2.83	17
PXDN‐LRR: NKG2C	1: 1	Q92626 P26717	40–245 116–231	1479 231	2.68	24
PXDN‐LRR: NKG2D^(MD)^	1: 1	Q92626 P26718	40–245 73–216	1479 216	2.01	24
PXDN‐LRR‐Ig: NKG2D	1: 1	Q92626 P26718	27–619 73–216	1479 216	15.78	13
PXDN‐LRR: OX40	1: 1	Q92626 P23510	40–245 51–183	1479 183	11.63	17
PXDN‐LRR: TNF10	1: 1	Q92626 P50591	40–245 39–263	1479 281	6.88	11
PXDN‐LRR: TNR4	1: 1	Q92626 P43489	40–245 29–214	1479 277	12.82	18
PXDN‐LRR: TNR9^(MD)^	1: 1	Q92626 Q07011	40–245 24–186	1479 255	1.10	24
PXDN‐LRR: TRAIL‐R2^(MD)^	1: 1	Q92626 O14763	40–245 56–210	1479 440	1.60	30

We further assessed complex stabilities in terms of binding energies using MM‐GB/SA calculations (Fig. 6D). Surprisingly, PXDN:TRAIL‐R2 showed the lowest binding energy (−30.8 ± 11.3 kcal·mol^−1^). Upon close inspection, we revealed that this binding energy is caused by two arginines (R186 and R188). In the complex structure, the two arginines are separated by the carboxylate group of a glutamate. During the MM‐GB/SA calculation, the glutamate gets removed leading to strong repulsive forces between the arginines, which artificially decreased the binding energy. Thus, we considered the low binding energies of PXDN:TRAIL‐R2 as not realistic but rather as an artifact of the one‐trajectory MM‐GB/SA approach. From the remaining interactions, the PXDN:NKG2D complex had the most favorable binding energy of −22.8 ± 7.7 kcal·mol^−1^, followed by PXDN:CD137 (−17.7 ± 8.4 kcal·mol^−1^), PXDN:TNR9 (−16.3 ± 7.4 kcal·mol^−1^), PXDN:CD70 (−14.4 ± 8.2 kcal·mol^−1^), and PXDN:CD48 (+3.3 ± 5.9 kcal·mol^−1^) (Fig. 6D).

Based on the high‐confidence structural model of the PXDN:NKG2D complex and its stability in unbiased MD simulations, we propose NKG2D as a relevant interaction partner for PXDN on NK cells. To gain further insights into this interaction, residue‐wise decomposition of binding energy identified key residues: R44 and R70 on the LRR domain form stable salt bridges with D115 and E201 on NKG2D, respectively (Fig. 6E,F). A hydrophobic patch (F43, I66, F71, L92), located between these two arginines on the LRR domain, interacts with hydrophobic residues on NKG2D (Y152, P206). Such hydrophobic patches surrounded by polar residues are frequently observed in protein–protein interfaces [63].

Overall, our modeling and simulation studies suggest that NKG2D interacts with the LRR domain of PXDN, consistent with our *in cellulo* findings that PXDN inhibits NK cell‐mediated anti‐melanoma cytotoxicity. This newly identified interaction may interfere with NKG2D activation or downstream signaling, thereby dampening NK cell responses against melanoma cells. Given the critical role of NKG2D in immune surveillance, our findings provide valuable insights into the immunomodulatory function of PXDN and its potential contribution to tumor immune evasion.

## 4 Discussion

Redox signaling is a well‐established driver of melanoma progression and cancer biology more broadly [64, 65, 66, 67], yet the precise molecular mechanisms underlying redox‐regulated immune evasion remain incompletely understood [68]. Recent advances, such as in ferroptosis research, have begun bridging the gap between redox biology and translational oncology [69, 70]. In this study, we aimed to uncover novel redox‐regulated mechanisms that contribute to melanoma malignancy and immune escape.

Using an unbiased, multi‐dataset transcriptomic approach, we identified a subset of redox‐associated genes potentially involved in melanoma progression. Among them, peroxidasin (PXDN), a secreted oxidoreductase previously linked to melanoma cell survival and extracellular matrix remodeling, emerged as a promising candidate.

However, initial functional analyses revealed that *PXDN* knockdown only modestly affected classical cancer hallmarks such as proliferation, invasion, and survival (Figs 1, 2, Figs S1–S3), prompting us to investigate alternative mechanisms.

Given PXDN's secretion and its potential to modulate the tumor microenvironment [38, 71], we hypothesized that PXDN may influence antitumor immunity, particularly the activity of natural killer cells—critical innate immune effectors whose importance in cancer immunotherapy is increasingly recognized [10, 72, 73, 74, 75]. Indeed, our findings provide the first evidence that PXDN interferes with NK cell‐mediated anti‐melanoma cytotoxicity. This aligns with previous studies showing that redox regulation can modulate NK cell function [74, 75] and supports the notion that PXDN acts beyond its canonical enzymatic roles.

Analysis of immunotherapy response datasets (TIGER) showed that *PXDN* expression is significantly elevated in non‐responders to both anti‐PD‐1 and anti‐CTLA‐4 therapies, and high *PXDN* levels correlate with worse patient survival (Fig. 3I–N). These observations suggest that PXDN may serve as a biomarker of immunotherapy resistance and contribute to immune evasion.

We further demonstrated that PXDN suppresses NK cell‐mediated killing (*pNKmK*) in a peroxidase‐independent manner. Addition of recombinant PXDN strongly reduced *pNKmK* in multiple melanoma lines, including PXDN knockdowns, and even in K562 leukemia cells (Fig. 4F–K), indicating a direct, tumor‐type–independent effect on NK cells. Rescue experiments via *PXDN* re‐expression and blockade with anti‐PXDN antibodies further confirmed the role of secreted PXDN in immune suppression (Fig. 4L–N).

Mechanistically, we found that PXDN does not alter expression of key NK ligands (e.g., *MICA/B*) in all contexts, suggesting an alternative mechanism. *In silico* modeling and molecular dynamics simulations identified a stable interaction between PXDN's leucine‐rich repeat domain and the NK cell receptor NKG2D. Among various receptor‐ligand simulations, the PXDN–NKG2D complex exhibited the most favorable binding energy and stability, supported by low PAE scores (Fig. 6). Arginine residues R44 and R70 on PXDN appeared critical for the interaction, forming salt bridges with negatively charged NKG2D residues (D115, E201), suggesting that the LRR domain may act as a competitive inhibitor of NKG2D activation.

Although mutational studies targeting this interface are conceptually appealing, they pose a risk of destabilizing PXDN's structural integrity. Instead, we propose an antibody‐based approach as a practical alternative, as supported by our results and prior data showing that anti‐NKG2D antibodies impair NK cell cytotoxicity [7].

Our findings place PXDN at the intersection of tumor immune evasion and redox regulation. While MD simulations alone cannot definitively prove a functional interaction, they support a plausible mechanism by which PXDN impairs NK cell surveillance through direct interaction with NKG2D. This complements prior evidence linking PXDN to immune modulation: one study proposed PXDN as an antagonist of IL‐1 receptor signaling in cytotoxic T lymphocytes [25], while another showed its N‐terminal domains bind lipopolysaccharides and mediate bacterial killing, implying a role in innate defense [76]. Together, these studies reinforce PXDN's broader immunoregulatory capacity.

In conclusion, our study identifies PXDN as a novel immune evasion factor in melanoma. We show that secreted PXDN suppresses NK cell‐mediated cytotoxicity independent of its redox activity, with impairment of NKG2D receptor activation as a proposed mode of action. These findings position PXDN as a key modulator of the melanoma immune microenvironment, with potential as a prognostic biomarker and therapeutic target. Targeting PXDN, either directly or through enhancement of NK cell responses, could complement existing checkpoint therapies and improve outcomes for patients with advanced melanoma. Moreover, this work contributes to the growing understanding of how redox enzymes influence immune evasion, opening new directions for immuno‐oncology research.

## 5 Conclusion

Our study identifies PXDN as a previously unrecognized regulator of melanoma immune evasion. While PXDN shows limited impact on classical tumor cell–intrinsic hallmarks, we demonstrate that secreted PXDN potently suppresses NK cell–mediated cytotoxicity in a peroxidase‐independent manner. Integrative transcriptomic analyses, functional assays, and molecular modeling converge on a mechanism in which PXDN interferes with NKG2D receptor activation, thereby weakening NK cell surveillance. Elevated PXDN expression in non‐responders to anti‐PD‐1 and anti‐CTLA‐4 therapy further highlights its clinical relevance and potential value as a biomarker of immunotherapy resistance.

Together, these findings position PXDN as a key modulator of the melanoma immune microenvironment and point to PXDN‐directed strategies or enhancement of NK cell function as promising avenues to complement existing checkpoint therapies.

## Conflict of interest

The authors declare no conflict of interest.

## Author contributions

Conceptualization: HMS, JM and IB; Investigation: HMS, DB, CI, DE, IST, CSG, LCMK, MS, JW, KW, JMP, AP, EB, WV, HS Formal analysis: HMS, DB, CI, DE, IST, CSG, LCMK, MS, JW, KW, JMP, EB, WV, HS; Funding acquisition: JM and IB; Project administration: JM and IB; Software and Resources: DB, WV, MG, HS, MPS; Supervision: HMS, MPS, JM and IB; Visualization: HMS, DB, CI, HS Writing—review & editing: all authors; Writing—original draft: HMS, JM and IB.

## Supporting information


**Fig. S1.** Impact of *PXDN* expression on melanoma progression.
**Fig. S2.** Genome and cell cycle characterization of PXDN‐depleted melanoma cell lines.
**Fig. S3.** 2D wound healing assay.
**Fig. S4.** Immunofluorescent, flow cytometry, and RNA sequencing analysis.
**Fig. S5.** Characterization of extracellular PXDN.
**Fig. S6.** AlphaFold2 models of PXDN.
**Fig. S7.** RMSD analysis of the simulations of PXDN complexes.
**Table S1.** Datasets for melanocyte vs melanoma DGE analysis.

## Data Availability

The computational data used for this study is available at the following publicly available repository: 10.5281/zenodo.15133144.
