# Supplementary material for: Peroxidasin enables melanoma immune escape by inhibiting natural killer cell cytotoxicity
Source: Mol Oncol. 2026 Jan 28;20(5):1161–84. doi: 10.1002/1878-0261.70191 (PMC13155146; doi:10.1002/1878-0261.70191)
Supplement: Supplementary file 1 — Fig. S1. Impact of PXDN expression on melanoma progression. Fig. S2. Genome and cell cycle characterization of PXDN‐depleted melanoma cell lines. Fig. S3. 2D wound healing assay. Fig. S4. Immunofluorescent, flow cytometry, and RNA sequencing analysis. Fig. S5. Characterization of extracellular PXDN. Fig. S6. AlphaFold2 models of PXDN. Fig. S7. RMSD analysis of the simulations of PXDN complexes. Table S1. Datasets for melanocyte vs melanoma DGE analysis. [file MOL2-20-1161-s001.pdf]

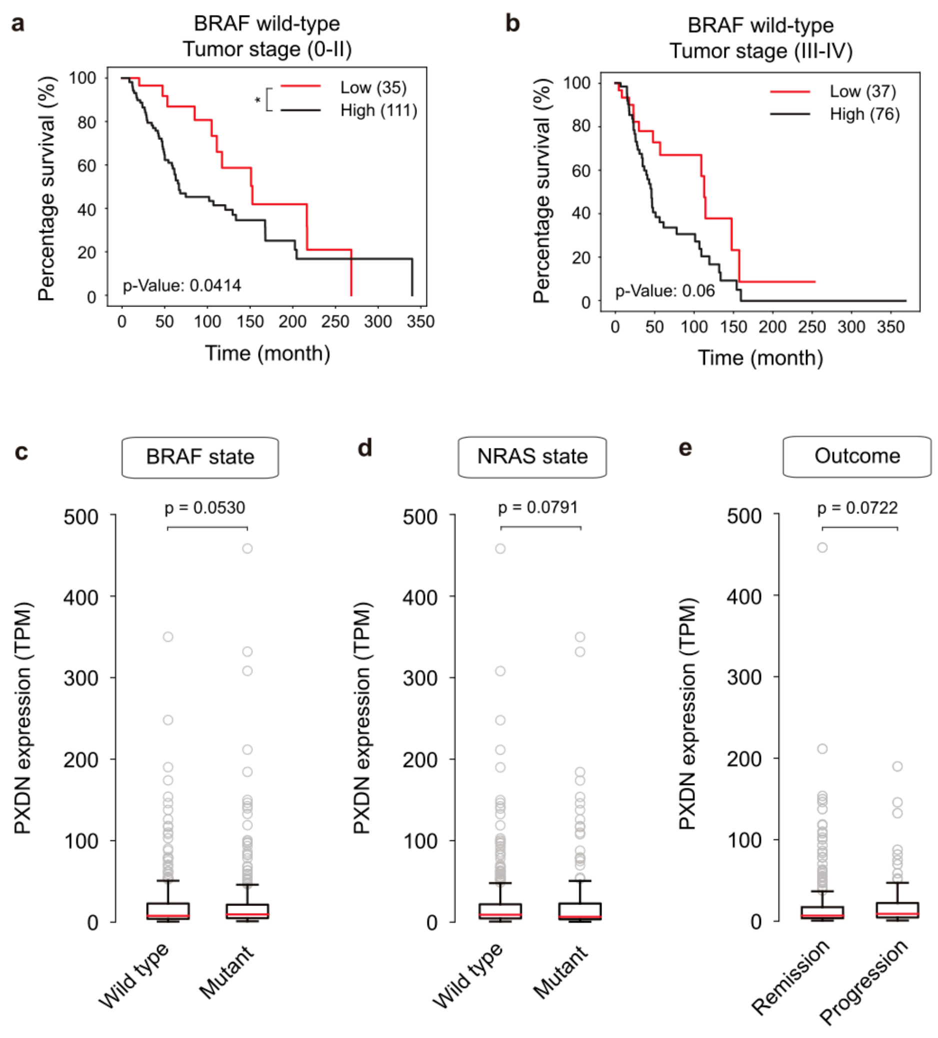

Figure S1: Impact of peroxidasin (PXDN) expression on melanoma progression

a-b) Kaplan–Meier survival estimation based on TCGA melanoma patient data from cBioPortal, comparing melanoma patients with wild-type BRAF and either high- (black) or low- PXDN (red) expression at different disease stages. **a)** tumor stage (0-II), **b)** tumor stage (III-IV). RNA sequencing data from TCGA was used to compare transcript per million reads (TPM) of PXDN mRNA between **c)** Melanoma patients with BRAF wild-type vs. BRAF mutant. **d)** Melanoma patients with NRAS wildtype vs. NRAS mutant. **e)** melanoma patients showing disease remission vs. disease progression. *p*-values calculated using a Mann-Whitney U test and log-rank test. *, $p < 0.05$.

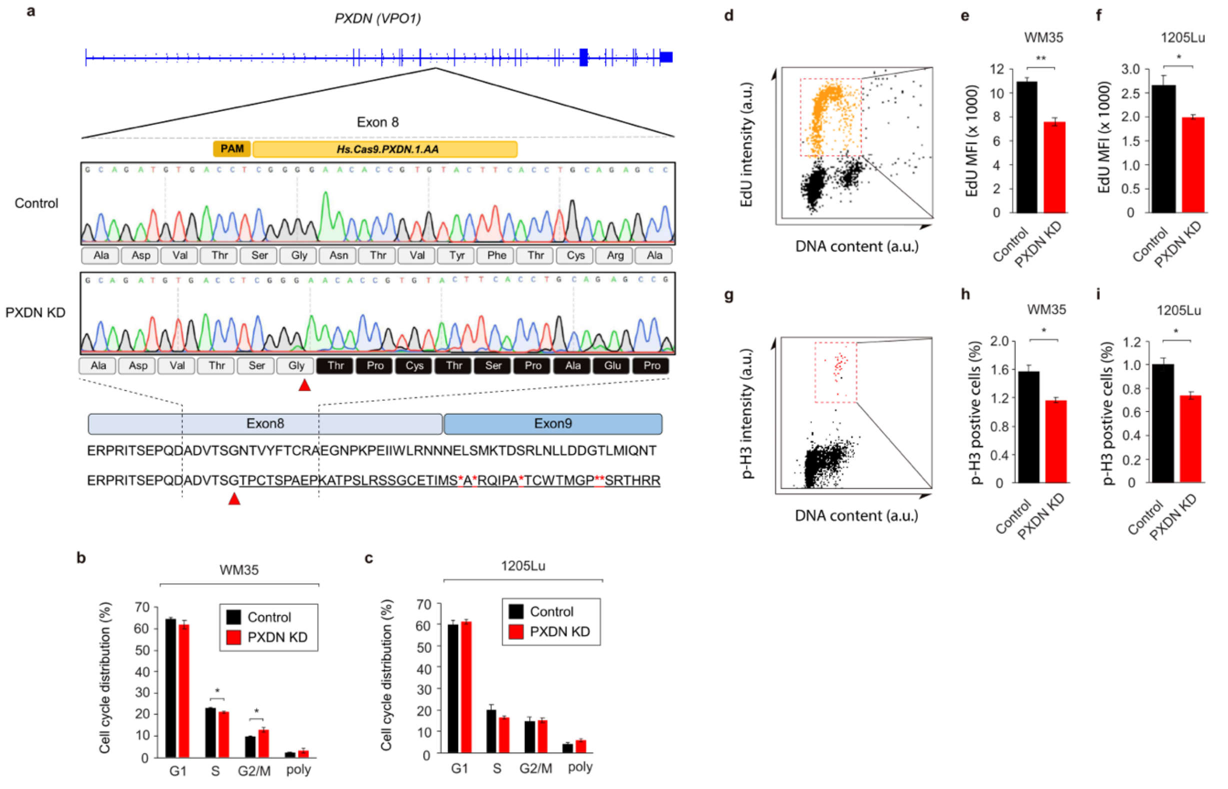

Figure S2: Genome and cell cycle characterization of PXDN-depleted melanoma cell lines
a) Genome configuration showing the target site of the guide RNA (Hs.Cas9.PXDN.1.AA), corresponding PAM motif, and coding region of targeted peroxidasin (PXDN) exon 8. Chromatograms of Sanger sequencing of the targeted region for control (upper) and PXDN KD (lower). Codon with indel is marked by red filled arrow; premature stop codons generated by CRISPR-Cas9 ribonucleoprotein (RNP) indicated by red asterisks. **b-c)** Cell cycle analysis. G1, G2/M and polyploidy populations determined by DNA content (2N, 4N, >4N respectively). S phase populations quantified by measuring EdU-positive cells. **d-f)** Quantification of EdU intensity. d) Representative image of the EdU analysis. EdU-positive cells in orange. Mean fluorescent intensity (MFI) of EdU signal in e) WM35 Control (black) and PXDN KD (red) and f) 1205Lu Control (black) and PXDN KD (red). **g-i)** Quantification of mitotic population. g) Representative image of FACS analysis detecting mitotic population. p-H3 positive cells in red. Percentage of mitotic cells in h) WM35 Control (black) and PXDN KD (red) and i) 1205Lu Control (black) and PXDN KD (red). Error bars indicate mean ± SEM; n = 4 independent experiments. *p*-values calculated using unpaired student t-test. ns = no significant difference, *, $p < 0.05$, **, $p < 0.01$.

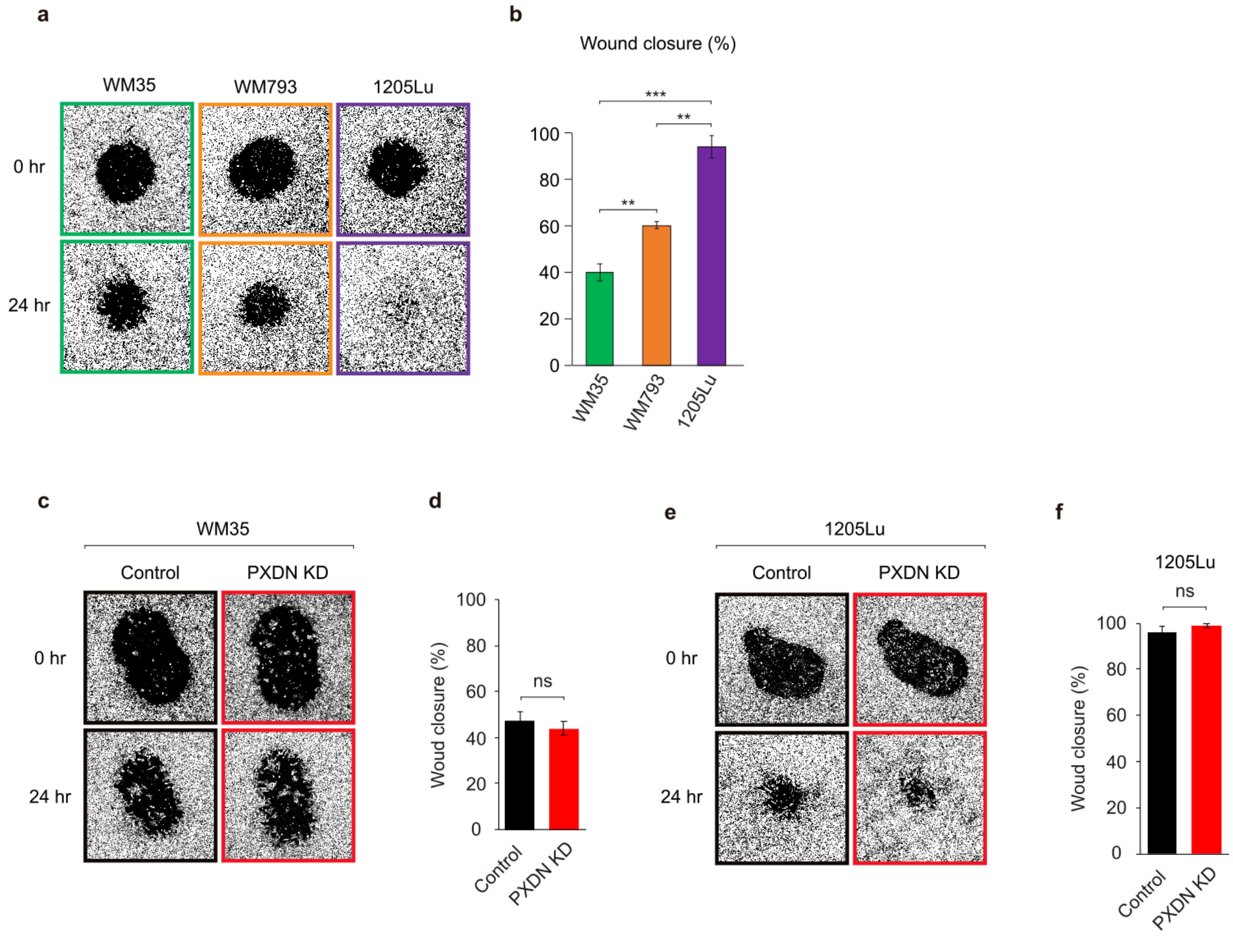

Figure S3: 2D wound healing assay

a-b) 2D wound healing assay by using melanoma cell lines of different disease phases. Green: Radial growth phase (RGP), Orange: Vertical growth phase (VGP), Violet: lung metastasis. **a)** Representative images of cell lines at 0 h (upper) and 24 h (bottom) using wound healing assay. **b)** Percentage of wound closure at 24 h after wound generation. Error bars indicate mean values ± SEM; WM35 (n = 22), WM793 (n = 29), 1205Lu (n = 24). *p*-values calculated using unpaired student t-test. $^{**}p < 0.01$, $^{***}p < 0.001$. **c-f)** 2D wound healing comparing control (black) and peroxidasin (PXDN) knockdown (KD) (red) in WM35 (c, d) and 1205Lu (e, f). **c, e)** Representative images of cell lines at 0 h (upper) and 24 h (bottom) in the wound healing assay. **d, f)** Percentage of wound closure at 24 h after wound generation. Error bars indicate mean values ± SEM; WM35 control (n = 30), WM35 PXDN KD (n = 30), 1205Lu control (n = 12), 1205Lu PXDN KD (n = 12). *p*-values calculated using unpaired student t-test. ns = no significant difference.

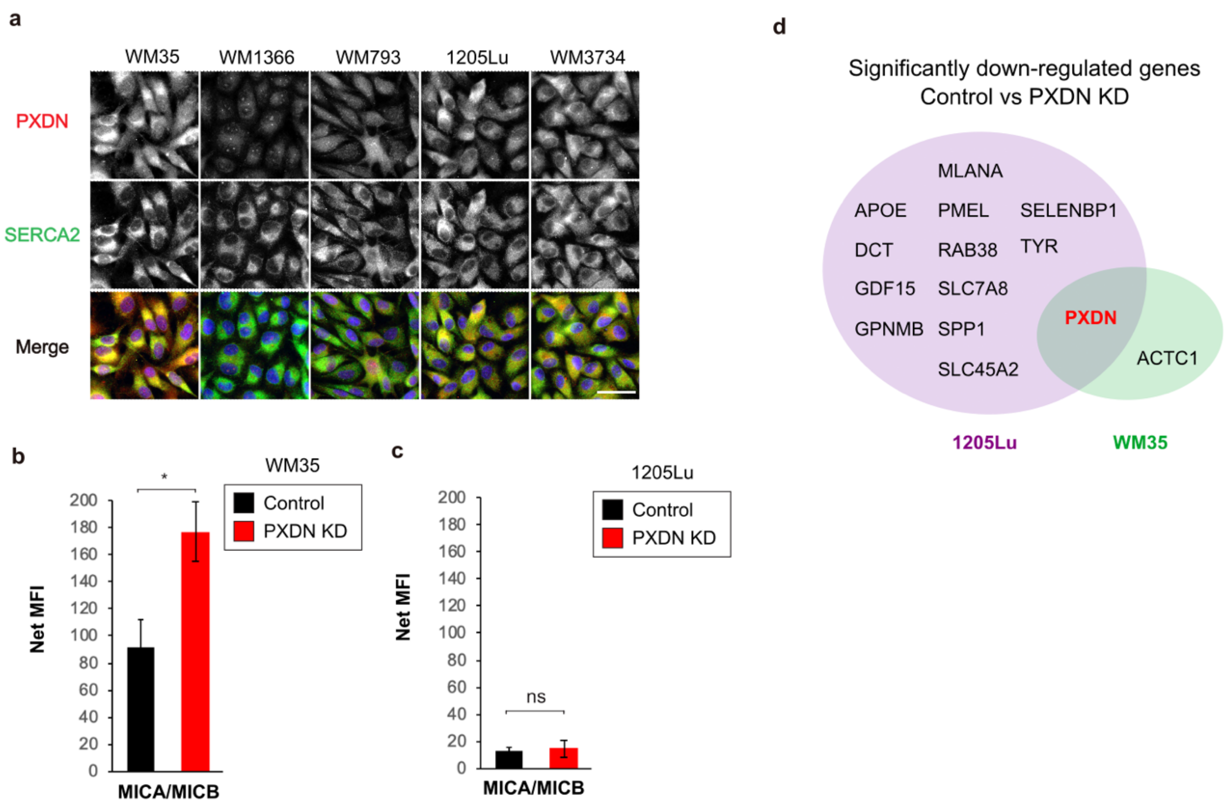

Figure S4: Immunofluorescent, flow cytometry, and RNA sequencing analysis
a) Representative images of immunofluorescent staining of peroxidasin (PXDN) (green) and Sarcoplasmic/Endoplasmic Reticulum Calcium ATPase 2 (SERCA2) (red) in indicated melanoma cell lines. Scale bar: 50 µm. **b-c)** Quantification of the net mean fluorescence intensity (MFI; minus MFI of corresponding isotype control) of MICA/MICB (MHC class I chain-related genes A/ B) in control (black) and PXDN knockdown (KD) (red) cells. **b)** WM35 (n = 4), **c)** 1205Lu (n = 7). *p*-values calculated using unpaired student t-test. ns = no significant difference, *p < 0.05. Error bars indicate mean values ± SEM. Differentially expressed genes (DEGs) between control and PXDN KD melanoma cells. **a)** Venn diagram showing the overlap of significantly down-regulated genes (Log2FC >1 and padj < 0.05) upon PXDN depletion between metastatic 1205Lu and primary WM35 cells.

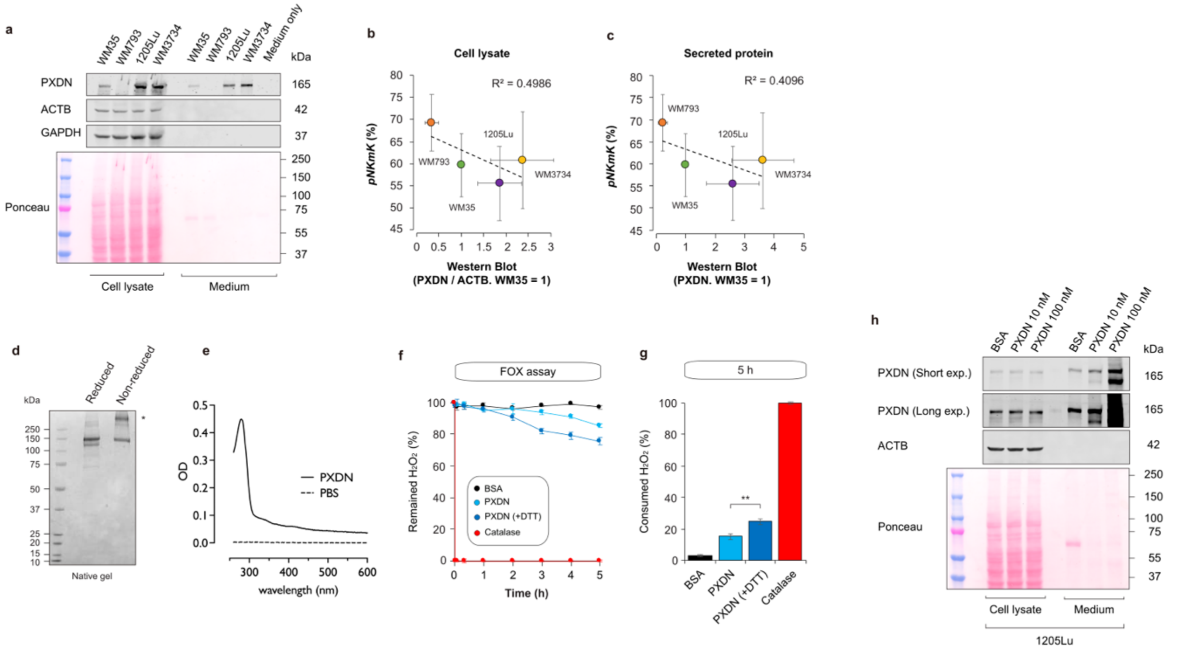

Figure S5: Characterization of extracellular peroxidasin (PXDN)

a) Representative image of Western blot detecting the intracellular and secreted PXDN in indicated melanoma cell lines. n = 3 independent experiments. **b-c**) Correlation between NK cell-mediated killing (*pNKmK*) and the expression of intracellular PXDN protein run on SDS-PAGE (b) and secreted PXDN protein (c). **b-c**) n = 3 independent experiments. Error bars indicate mean values ± SEM. **d)** Representative image of recombinant PXDN run under native gel electrophoresis. Reduced PXDN proteins were treated with dithiothreitol (DTT). Covalently-linked PXDN marked by an asterisk. **e)** PXDN does not exhibit the usual heme Soret peak around 420 nm. Visible absorption spectrum of 0.7 mg/ml PXDN in phosphate-buffered saline (PBS) shown. **f-g)** Ferrous Oxidation in Xylenol orange (FOX) assay monitoring peroxidase activity of recombinant PXDN. Catalase used as positive control. **f)** Kinetics of H_2O_2 consumption during FOX assay. **g)** Quantification of the consumed H_2O_2 after 5 h of FOX assay. n = 3 independent experiments. *p*-values calculated using unpaired student t-test. $^{**}p < 0.01$. **h)** Representative image of Western blot showing intracellular and extracellular pools of PXDN upon addition of recombinant PXDN. Recombinant PXDN added to cell culture medium 24 h prior to protein precipitation. Final concentration of 100 nM bovine serum albumin (BSA) added as control for protein precipitation. *β*-Actin (ACTB) and glyceraldehyde-3-phosphate dehydrogenase (GAPDH) used as controls for intracellular proteins.

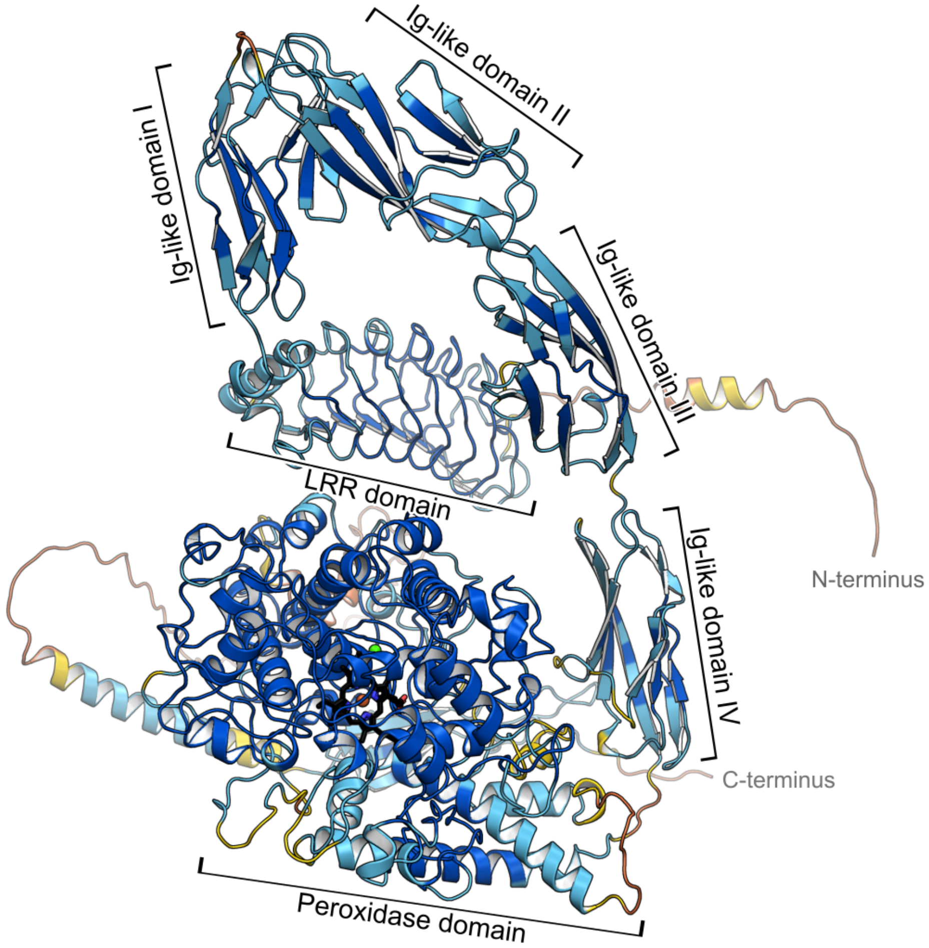

Figure S6: AlphaFold2 model of PXDN
A full-length model of PXDN with labelled folded domains. The model is colored according to the predicted local distance different test (pLDDT) score: dark blue [90, 100], light blue [70, 90), yellow [50, 70), and orange [0, 50).

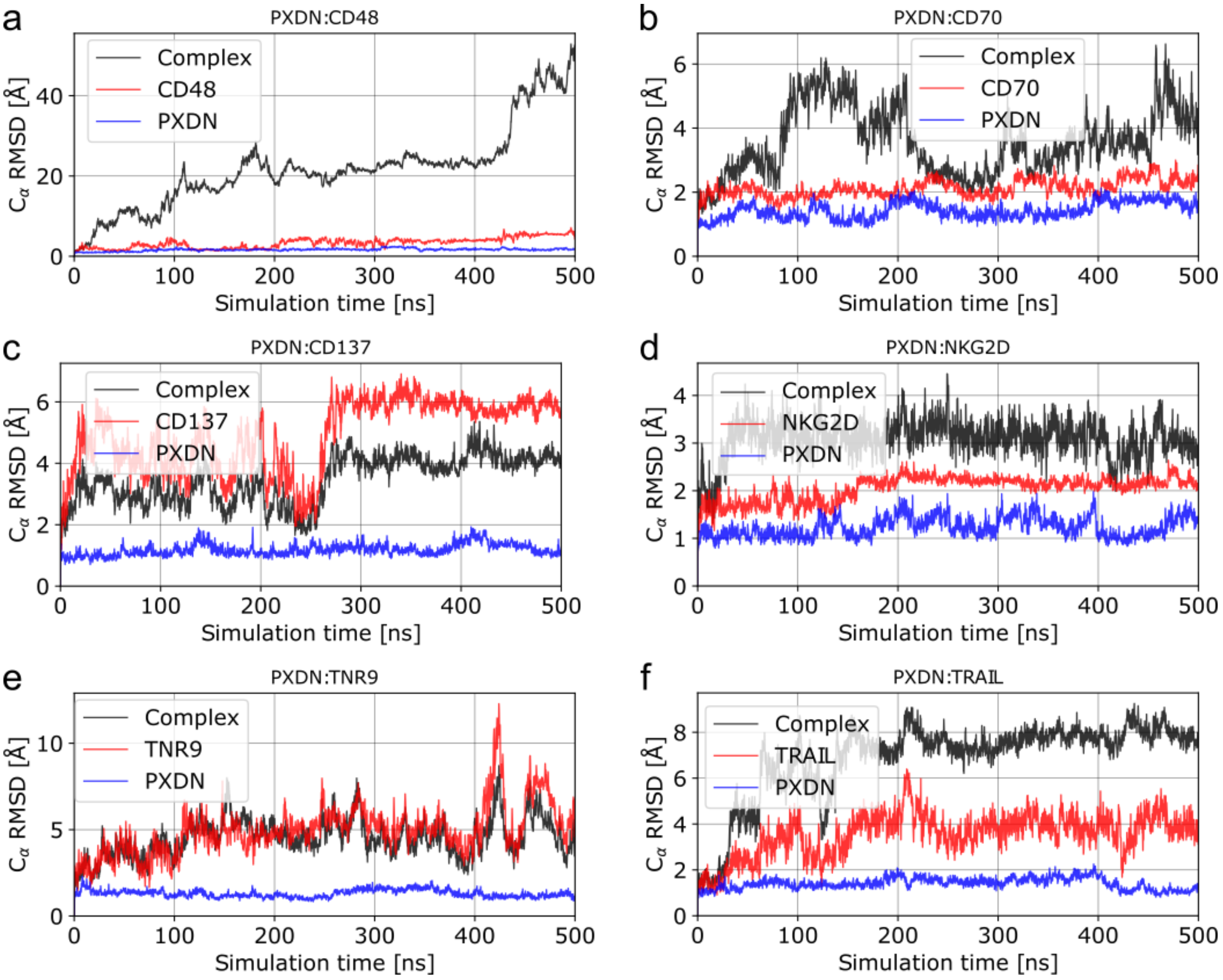

Figure S7: RMSD analysis of the simulations of PXDN complexes.
The root mean square deviation (RMSD) was calculated for all simulations with respect to an initial energy minimized structure. The RMSD of the individual leucine-rich repeats (LRRs) of PXDN, the NK cell/melanoma receptor (red), and the whole complex (black) are shown. **a)** PXDN:CD48. **b)** PXDN:CD70. **c)** PXDN:CD137. **d)** PXDN:NKG2D. **e)** PXDN:TNR9. **f)** PXDN:TRAIL-R2.

Table S1: Datasets for Melanocyte vs Melanoma DGE analysis

	Datasets for Melanocyte vs Melanoma DGE analysis		
	**Accession number**	**Publication PMID**	**Number of DGE profiles**
	GSE123686	30641895	3
	GSE31879	NA	1
	GSE111766	NA	5
	GSE83583	27545456	1
	GSE86373	NA	2
	GSE62075	25746835	3
	GSE4570	15289333	1
	GSE65568	28234767	1
	GSE44662	24014427	1
	GSE31909	NA	2
	GSE46517	20520718	1
	GSE22301	21343389	1
	GSE7553	18442402	1
	GSE4587	16251803	1
	GSE15281	NA	2
	GSE122907	30719424	3
**Total No.of DGE profiles**			**29**
